# UAV-based intelligent traffic surveillance using recurrent neural networks and Swin transformer for dynamic environments

**DOI:** 10.3389/fnbot.2025.1681341

**Published:** 2025-10-13

**Authors:** Mohammed Alshehri, Ting Wu, Nouf Abdullah Almujally, Yahya AlQahtani, Muhammad Hanzla, Ahmad Jalal, Hui Liu

**Affiliations:** ^1^Department of Computer Science, King Khalid University, Abha, Saudi Arabia; ^2^Department of Otorhinolaryngology Head and Neck Surgery, Nanjing Tongren Hospital, School of Medicine, Southeast University, Nanjing, China; ^3^Department of Information System, College of Computer and Information Sciences, Princess Nourah bint Abdulrahman University, Riyadh, Saudi Arabia; ^4^Department of Informatics and Computer Systems, King Khalid University, Abha, Saudi Arabia; ^5^Department of Computer Science, Air University, Islamabad, Pakistan; ^6^Department of Computer Science and Engineering, College of Informatics, Korea University, Seoul, Republic of Korea; ^7^Jiangsu Key Laboratory of Intelligent Medical Image Computing, School of Artificial Intelligence (School of Future Technology), Nanjing University of Information Science and Technology, Nanjing, China; ^8^Guodian Nanjing Automation Co., Ltd., Nanjing, China; ^9^Cognitive Systems Lab, University of Bremen, Bremen, Germany

**Keywords:** neural networks, unmanned aerial vehicle, multi-object tracking, adaptive control, swin transformer, autonomous systems

## Abstract

**Introduction:**

Urban traffic congestion, environmental degradation, and road safety challenges necessitate intelligent aerial robotic systems capable of real-time adaptive decision-making. Unmanned Aerial Vehicles (UAVs), with their flexible deployment and high vantage point, offer a promising solution for large-scale traffic surveillance in complex urban environments. This study introduces a UAV-based neural framework that addresses challenges such as asymmetric vehicle motion, scale variations, and spatial inconsistencies in aerial imagery.

**Methods:**

The proposed system integrates a multi-stage pipeline encompassing contrast enhancement and region-based clustering to optimize segmentation while maintaining computational efficiency for resource-constrained UAV platforms. Vehicle detection is carried out using a Recurrent Neural Network (RNN), optimized via a hybrid loss function combining cross-entropy and mean squared error to improve localization and confidence estimation. Upon detection, the system branches into two neural submodules: (i) a classification stream utilizing SURF and BRISK descriptors integrated with a Swin Transformer backbone for precise vehicle categorization, and (ii) a multi-object tracking stream employing DeepSORT, which fuses motion and appearance features within an affinity matrix for robust trajectory association.

**Results:**

Comprehensive evaluation on three benchmark UAV datasets—AU-AIR, UAVDT, and VAID shows consistent and high performance. The model achieved detection precisions of 0.913, 0.930, and 0.920; tracking precisions of 0.901, 0.881, and 0.890; and classification accuracies of 92.14, 92.75, and 91.25%, respectively.

**Discussion:**

These findings highlight the adaptability, robustness, and real-time viability of the proposed architecture in aerial traffic surveillance applications. By effectively integrating detection, classification, and tracking within a unified neural framework, the system contributes significant advancements to intelligent UAV-based traffic monitoring and supports future developments in smart city mobility and decision-making systems.

## 1 Introduction

Recent breakthroughs in deep learning, transformer architectures, and attention-driven vision models have substantially advanced object detection, classification, and multi-object tracking in complex visual environments (Sundaresan et al., 2024; Zhang et al., 2025). These algorithmic advances underpin modern autonomous systems and intelligent transportation infrastructures by enabling improved vehicle identification and behavior analysis under varying conditions (Qureshi et al., 2023). Nevertheless, traditional ground-based traffic surveillance relies on fixed cameras and infrastructure suffers from occlusion, limited spatial coverage, and low adaptability to diverse urban layouts (Vu et al., 2025; Mei and Zhu, 2024; Hanzla and Jalal, 2025). Such constraints limit their usefulness for large-scale, flexible monitoring required by smart-city and emergency-response applications.

Unmanned Aerial Vehicles (UAVs) provide a complementary sensing modality that addresses many of these limitations by offering mobile, high-resolution observation with dynamic field-of-view control (Hanzla et al., 2024). When combined with deep learning and reinforcement-based control strategies, UAVs enable scalable video understanding and adaptive data collection for traffic monitoring. At the same time, aerial imagery imposes unique technical challenges cluttered backgrounds, irregular object scales, asymmetric spatial distributions, and dynamic motion which complicate detection and raise computational demands (Ammour et al., 2017). These factors motivate solutions that are both robust to visual artifacts and efficient enough for onboard or near-edge operation.

In response, we present a symmetry-aware UAV traffic monitoring framework that explicitly targets the principal challenges of aerial surveillance. The pipeline begins with Fast Adaptive Mean-Variance Normalization (FAMVN) to reduce illumination bias and background effects, followed by spectral–spatial segmentation (SASSC) to sharpen object boundaries and suppress clutter (Mujtaba et al., 2024a,b; Almujally et al., 2024). To exploit temporal continuity in videos, we employ an RNN-based temporal detector with attention mechanisms that improve bounding-box alignment and reduce transient false positives. Detected candidates are processed in parallel by (i) DeepSORT for identity-preserving trajectory estimation and (ii) a classification branch that fuses handcrafted descriptors (SURF + BRISK) with a Swin Transformer to handle scale variability and class imbalance. This dual-path design leverages complementary strengths preprocessing and segmentation to improve proposals, temporal modeling to enforce detection continuity, and hybrid feature fusion to boost classification reliability yielding robust detection, tracking stability, and improved classification for aerial sequences.

Key contributions of this work are:

- Traditional background subtraction and elimination techniques are ineffective for aerial imagery due to continuously changing and dynamic backgrounds. To overcome this limitation, we adopted the Self-Adaptive Spectral-Spatial Clustering (SASSC) algorithm, which clusters pixels with high spectral-spatial correlation, effectively isolating relevant regions.
- For vehicle detection, we employed a Recurrent Neural Network (RNN), a temporal deep learning architecture well-suited for modeling sequential dependencies across video frames, enabling accurate identification of small-scale vehicles in aerial views where objects appear tiny, occluded, and densely clustered.
- The system is designed to support both vehicle tracking and classification. Moving and stationary vehicles are first distinguished to ensure only dynamic vehicles are tracked. DeepSORT is used as the tracking module, leveraging both motion and appearance features to maintain consistent identities across frames.
- Simultaneously, each detected vehicle undergoes feature extraction. The extracted features are then processed through a Swin Transformer, which classifies each vehicle into its respective category with high accuracy.

The remainder of the paper is structured as follows: Section 2 presents related work; Section 3 explains the system architecture; Section 4 discusses experimental results and comparisons; Section 5 provides a detailed discussion; and Section 6 concludes the study and outlines future research directions.

## 2 Literature review

Effective road traffic monitoring has been a critical area of research, with numerous approaches proposed to enhance vehicle detection and tracking. This section provides a comprehensive overview of recent advancements, highlighting key methodologies, challenges, and emerging trends in the field.

### 2.1 Traditional methods in vehicle detection and tracking

(Yang and Qu, 2018)introduced a real-time surveillance framework leveraging a background subtraction model based on low-rank and sparse decomposition to detect moving vehicles, followed by an online Kalman filter for object tracking and counting. This classical pipeline demonstrates high robustness in complex scenes involving overlapping vehicles or visual clutter (Moutakki et al., 2017) developed a vehicle counting system using a codebook-based background model with occlusion handling. Vehicle regions are segmented via background subtraction, refined through contour filtering, and classified using HOG descriptors and an SVM, achieving comprehensive detection accuracy on highway data (Nosheen et al., 2024) proposed a lightweight approach combining blob detection with a Kernelized Correlation Filter (KCF) tracker. The system applies preprocessing (gamma correction, bilateral filtering, and mean-shift) to enhance vehicle blobs, which are then tracked using KCF, achieving around 82% detection and 86% tracking accuracy on the KITTI dataset. These studies highlight the continued relevance of classical methods such as Kalman and KCF tracking in real-time surveillance. Many other approaches utilize sequential filtering (e.g., Gaussian blur, morphological operations) or optical flow, followed by simple trackers. Traditional background subtraction models like Codebook or Mixture of Gaussians, when paired with contour filtering and trackers (e.g., Kalman, Mean-Shift, or particle filters), remain computationally efficient and interpretable, though they may degrade under severe occlusion or dynamic lighting.

### 2.2 Machine learning-based traffic scene analysis

Arinaldi et al. (2018) compared a classical Mixture-of-Gaussians (MoG) background model paired with HOG features and an SVM classifier against a deep learning-based Faster R-CNN. While MoG+SVM effectively segmented and classified vehicles, it under-performed in scenarios with occlusions or stationary vehicles, where Faster R-CNN proved more robust. ElSahly and Abdelfatah (2023) developed a Random Forest (RF)-based traffic incident detection system using VISSIM-generated simulations incorporating con-gestion, incident severity, and sensor data. The RF classifier accurately distinguished between incident and normal traffic states, demonstrating the value of classical ML in high-level anomaly detection. Other ML-based approaches leverage hand-crafted features such as HOG or Haar cascades with SVMs or boosting to estimate traffic flow, classify vehicle types, or detect static anomalies. Though computationally efficient, these methods often suffer from limited robustness under viewpoint or illumination changes.

Alharbi (2022) applied RF for vehicle detection in UAV imagery, achieving 87.4% recall under challenging conditions but facing performance drops with dense traffic and high-dimensional features. Meanwhile, Chandramohan et al. (2020) addressed the traffic surge in urban environments, emphasizing the importance of efficient vehicle clustering in VANETs to reduce redundant communication, maintain data integrity, and support real-time traffic management. Wang et al. (2022) address the critical challenge of maximizing fresh information collected by Unmanned Aerial Vehicles (UAVs) from fixed-point devices within complex forest environments, a task traditionally handled by human patrols but better suited for UAVs despite the limitations of existing path planning methods in such intricate settings. They propose two distinct methodologies: for two-point path planning, they employ a chaotic initialization and co-evolutionary algorithm, carefully considering key UAV performance and environmental factors and for multi-point path planning, they introduce a method based on simulated annealing. Experimental validation, which involved using benchmark functions for parameter configuration and comparing their approach against existing strategies on both simple and complex simulated maps, demonstrated that their proposed techniques effectively generate UAV patrol paths that achieve higher information freshness with fewer iterations and lower computational costs, thereby underscoring their practical value. Bianchi et al. (2024) proposed an energy-optimal reference generator combined with a hierarchical control strategy for quadrotor trajectory planning. Their approach explicitly minimizes energy expenditure while preserving system stability, showing that real-time feasible control can be achieved with substantial reductions in energy consumption. By optimizing trajectory references at the control level, the framework provides a foundation for energy-conscious UAV deployment, particularly valuable for long-duration or resource-limited aerial missions. This study illustrates how energy considerations can be embedded directly into UAV control architectures for efficient real-world operations.

### 2.3 Deep learning-based methods

The emergence of deep learning has significantly advanced vehicle detection, classification, and tracking. One-stage detectors like YOLO, particularly YOLOv3 and its successors, have become widely adopted for real-time applications due to their balance between speed and accuracy. However, YOLOv3 struggles with occluded or small-scale objects. Two-stage models such as Faster R-CNN, when integrated with tracking modules like Kalman filters and the Hungarian algorithm (as in the SORT framework), provide better multi-object tracking in dynamic scenes, though they lack appearance-based discrimination, which affects performance in dense traffic.

Gallo et al. (2023) employed YOLOv7 for UAV-based detection, achieving strong precision for small objects, albeit at the cost of high computational demand, limiting deployment on edge devices. Improvements such as k-means clustering for anchor box generation and multi-scale feature fusion have further enhanced earlier YOLO versions; for instance, Sang et al. (2018) proposed BIT-Vehicle dataset using an optimized YOLOv2 model. Similarly, YOLOv5 has demonstrated superior accuracy over YOLOv3/4, especially on aerial datasets, as noted by Nepal and Eslamiat (2022). Recent trends include ensemble models combining EfficientDet and YOLO variants to improve robustness under occlusion and scale variation. Transformer-based detectors and networks like YOLOv8 continue to push performance boundaries, while two-stage models such as Mask R-CNN provide fine-grained segmentation for precise vehicle shape extraction in cluttered scenes. Overall, deep CNN-based detectors both single-stage and two-stage remain the foundation of modern vehicle detection pipelines, consistently achieving state-of-the-art performance on benchmarks like KITTI, UA-Detrac, and UAVDT.

Battal et al. (2023) used YOLOv5m6 on real-world traffic video sequences to detect and classify five vehicle categories, reporting an average detection/classification accuracy of 88% across diverse conditions. Xu H. et al. (2020); Xu M. et al. (2020) introduced an improved multitask-cascaded CNN (IMC-CNN) with mixed image enhancement techniques for aerial vehicle detection; although they improved small-object recall, the detection precision plateaued at 85% and Biyik et al. (2023) trained standard YOLOv3 and YOLOv4-CSP models on orthophotos from UAV imagery; they reported mAPs of 80 and 87%, respectively, underscoring the difficulty of detection in high-noise geo-referenced contexts. Collectively, these results illustrate that while current deep architectures can approach the lowest 90s in accuracy, they often remain below 91%, especially in outdoor, aerial, or real-time video scenarios further motivating the refined approach and higher scores (≥0.92) achieved in our proposed system across AU-AIR, UAVDT, and VAID

### 2.4 Challenges in existing work

Despite significant progress in aerial traffic monitoring, several critical challenges limit the deployment of current methods on real-world, edge-based platforms. Traditional approaches often rely on static background modeling, which proves inadequate for UAV imagery, where dynamic perspectives and shifting backgrounds are the norm. The detection of small-scale vehicles remains problematic due to resolution constraints and occlusion, especially in cluttered environments. Moreover, many state-of-the-art deep learning models are computationally intensive, making them unsuitable for real-time inference on re-source-constrained, low-power UAV systems [6]. This severely impacts the scalability and cost-effectiveness of aerial surveillance in smart cities. Additionally, existing methods struggle to distinguish between moving and stationary vehicles, particularly under dense traffic and shadowed regions, leading to errors in trajectory tracking and event recognition. Class imbalance in datasets where dominant vehicle types overshadow minority classes further introduces bias, compromising classification reliability. These limitations highlight the urgent need for lightweight, energy-efficient, and edge-deployable solutions tailored for UAV platforms to ensure real-time, accurate, and scalable traffic intelligence in complex aerial scenarios.

## 3 Materials and method

### 3.1 System methodology

**Figure 1:**
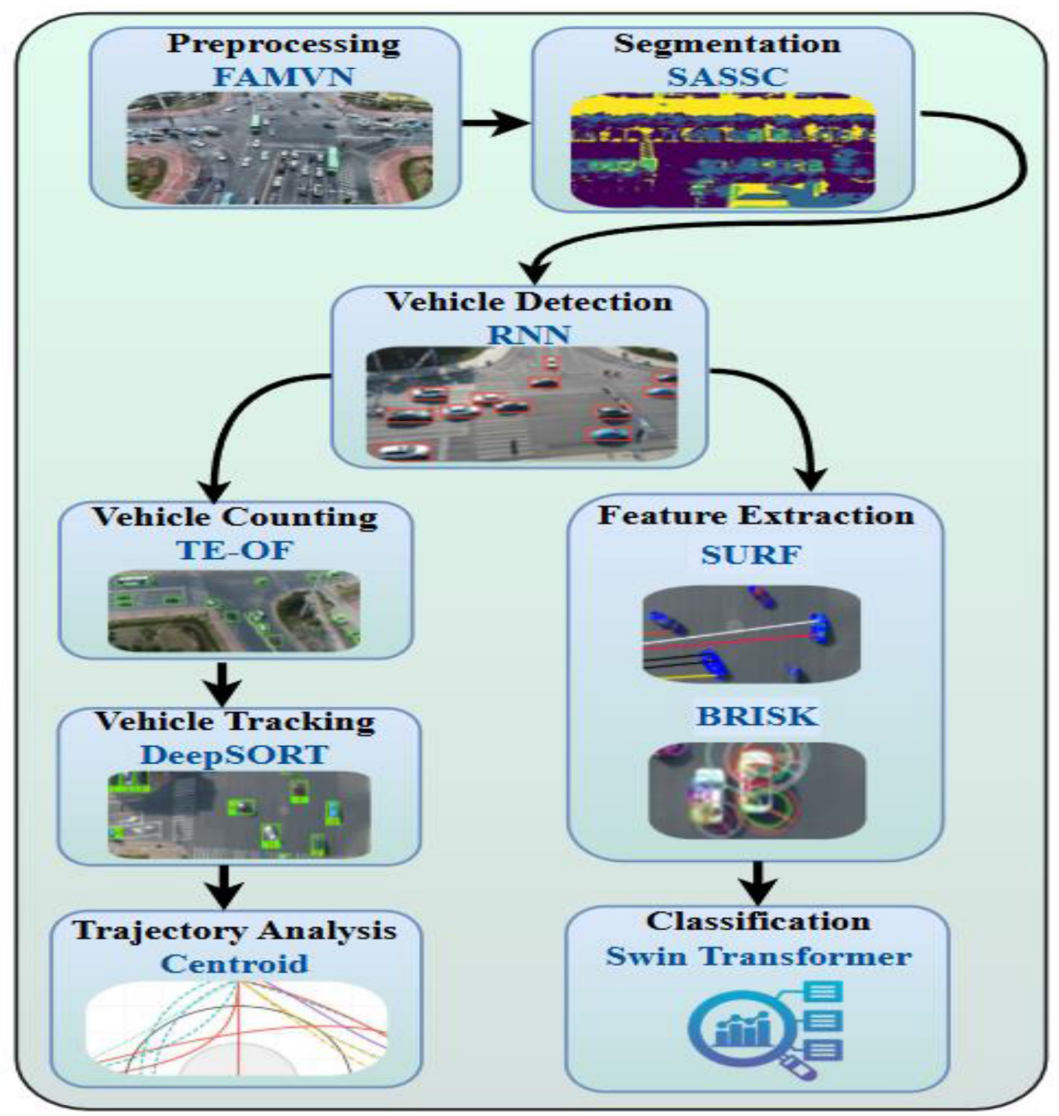
Architecture of the UAV-based intelligent traffic surveillance system integrating deep learning and adaptive control for real-time vehicle detection and classification in urban environments.

The novelty of the proposed approach lies in the systematic integration of multiple complementary methods into a single pipeline. Figure 1 illustrates this integration scheme: (i) preprocessing with FAMVN and contextual smoothing reduces illumination bias and noise, providing clean inputs for segmentation; (ii) segmentation with SASSC enforces spectral–spatial coherence, improving object boundary preservation; (iii) temporal RNN-based detection leverages motion continuity to reduce false positives; (iv) DeepSORT maintains consistent object identities across frames; (v) SURF and BRISK descriptors are fused to capture both scale-robust and illumination-robust features; and (vi) the Swin Transformer performs final classification by exploiting hierarchical self-attention to combine local and global cues. The combined use of these techniques enhances robustness by compensating for the limitations of any single method—for example, DeepSORT corrects missed detections from the RNN, while feature fusion strengthens classification under challenging aerial conditions. This synergistic integration directly contributes to improved detection precision, tracking stability, and classification accuracy, thereby advancing the feasibility of UAV-based real-time surveillance.

### 3.2 Image preprocessing via fast adaptive mean-variance normalization (FAMVN)

To improve data quality across the AU-AIR, VAID, and UAVDT datasets, we implemented an adaptive preprocessing pipeline integrating classical and advanced enhancement techniques (Mujtaba et al., 2024a,b). Rather than relying on traditional histogram methods, we applied Fast Adaptive Mean-Variance Normalization (FAMVN), which preserves structural features under varying lighting conditions. Frames 32,823 (AU-AIR), 6,000 (VAID), and 80,000 (UAVDT) were resized to 640 × 640 and pixel values normalized to [0,1] for model stability. Unlike CLAHE, FAMVN adjusts both local mean and variance within overlapping kernels, enhancing contrast while preserving edges and minimizing over-saturation. The FAMVN transformation for a pixel *x*_*i, j*_ in window *w*_*i, j*_ is defined as:


(1) $$I_{i,j}^{\prime }=\frac{I_{i,j}-\mu w_{i,j}}{\sigma w_{i,j}\;+\in }.\;\sigma_{T}+\mu_{T}$$

**Figure 2:**
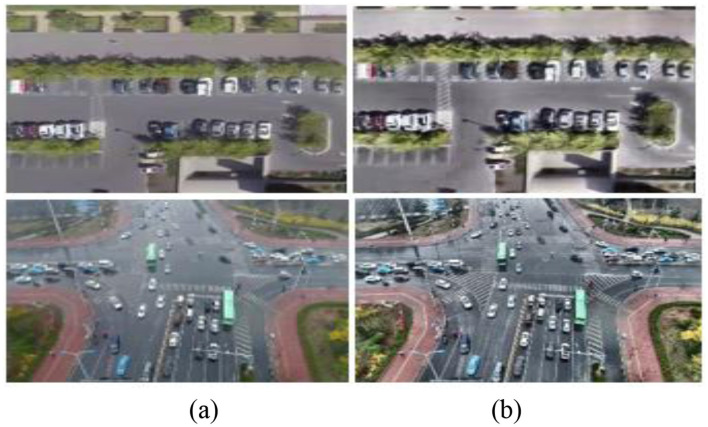
Preprocessed images **(a)** original images **(b)** Enhanced images via FAMVN.

Here, **μ*w***_***i,j***_ and **σ*w***_***i,j***_ denote the local mean and standard deviation within the sliding window ***w***_***i,j***_, while ***μ***_***T***_ and ***σ***_***T***_ represent the global dataset statistics used as normalization targets. The small constant ϵ ensures numerical stability during division. This normalization aligns local intensity variations with global distribution statistics, thereby reducing illumination bias and enhancing structural consistency across frames. To further preserve edge information while suppressing noise, we apply Multiscale Contextual Smoothing using a Gaussian-integrated bilateral filter, which maintains salient object boundaries critical for robust detection and tracking in aerial imagery, as shown in Figure 2.

### 3.3 Image segmentation via self-adaptive spectral-spatial clustering (SASSC)

Following preprocessing, segmentation was performed using Self-Adaptive Spectral-Spatial Clustering (SASSC), an unsupervised method that combines spectral intensity patterns with spatial continuity. Unlike traditional Fuzzy C-Means (FCM), which lacks spatial awareness, SASSC employs graph-based manifold learning and adaptive neighborhood consensus to refine cluster memberships. This dual-domain strategy enhances boundary preservation and noise robustness key for accurate aerial vehicle segmentation:


(2) $$\begin{array}{l} \mu_{ij}^{\left(t+1\right)}=\frac{\emptyset_{ij}^{\beta }.\omega_{ij}^{\gamma }}{\overset{C}{\underset{k=1}{\sum }}\emptyset_{kj}^{\beta }.\omega_{kj}^{\gamma }}\;for\;i=1,\ldots ,C;j=1,\ldots ,N \end{array}$$

Here, φ_*ij*_ quantifies the spectral similarity between pixel *x*_*j*_ and cluster center *v*_*i*_, while ω_*ij*_ measures spatial consistency by encoding the proportion of neighboring pixels of *x*_*j*_ that are assigned to cluster *i*. The exponents β and γ\ balance these two influences, allowing the update to emphasize either spectral fidelity (β) or spatial smoothness (γ). The denominator ensures probabilistic normalization across all clusters, so that the memberships $\mu_{ij}^{\left(t+1\right)}$ remain valid probabilities. This formulation thus integrates local neighborhood structure into the clustering process, yielding assignments that are both spectrally representative and spatially coherent. As a result, the method becomes more robust to edge noise and background clutter in aerial imagery. Convergence is guaranteed through an adaptive entropy-based stopping criterion, which halts iterations once successive cluster centers stabilize within a defined threshold:


(3) $$\begin{array}{l} \left(t\right)=\overset{C}{\underset{i=1}{\sum }}H\left(v_{i}^{\left(t\right)}\right).||v_{i}^{\left(t\right)}-v_{i}^{\left(t-1\right)}|{|}^{2}<\in \end{array}$$

**Figure 3:**
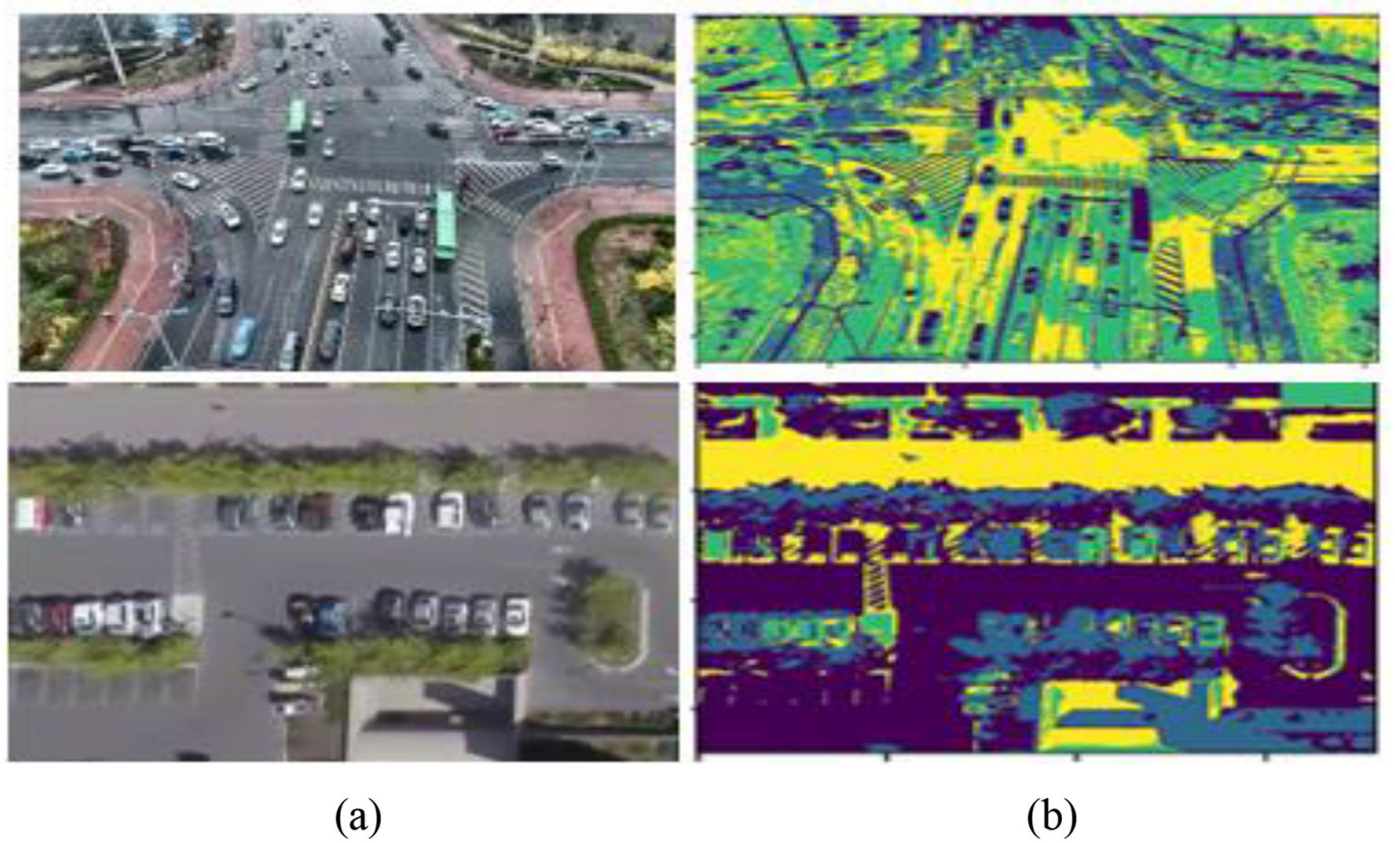
Segmentation of images using SASSC **(a)** original images **(b)** segmented images.

Here, $H\left(v_{i}^{\left(t\right)}\right)$ denotes the fuzzy entropy of cluster *i* at iteration *t*, which measures the uncertainty in its membership distribution. The term $||v_{i}^{\left(t\right)}-v_{i}^{\left(t-1\right)}|{|}^{2}$ quantifies the squared change in the cluster center between consecutive iterations, indicating how much the cluster is shifting. By weighing this change with the entropy, clusters with higher uncertainty exert a stronger influence on the stopping criterion. Summation across all *C* clusters provides a global measure of stability for the clustering process. Convergence is declared once this entropy-weighted variation falls below the predefined threshold ϵ, ensuring that the algorithm halts only when both stable and uncertain clusters have sufficiently settled. This prevents premature convergence in highly dynamic or noisy regions, leading to more reliable segmentation results, as illustrated in Figure 3.

Unlike traditional methods such as CLAHE, which relies on local histogram equalization, FAMVN performs adaptive mean-variance normalization with Gaussian-integrated bilateral filtering. This design explicitly preserves edges while suppressing background illumination artifacts, making it more suitable for UAV imagery. Similarly, SASSC differs from classical Fuzzy C-Means by introducing a joint spectral–spatial weighting scheme, controlled by parameters β and γ, and by adopting an entropy-based adaptive stopping criterion. In our experiments, window size = 15 × 15, β = 2, and γ = 1 provided the best balance between noise suppression and object coherence.

### 3.4 Vehicle detection via recurrent neural networks (RNN)

Recurrent Neural Networks (RNNs), especially their advanced variants like Long Short-Term Memory (LSTM) and Gated Recurrent Unit (GRU), are highly effective for modeling temporal sequences in aerial video analysis. While conventional object detectors (e.g., YOLO, Faster R-CNN) focus on spatial detection per frame, they often lack the temporal awareness necessary for tracking and detecting fast-moving or intermittently visible vehicles across successive frames. This is particularly challenging in aerial surveillance, where dynamic environmental factors such as occlusions, shadows, scale variation, and abrupt viewpoint changes can degrade detection accuracy.

To address this, we propose integrating RNNs as temporal feature aggregators following the spatial feature extraction stage. Frame-wise feature embeddings, obtained using a deep convolutional backbone (e.g., ResNet50 or CSPDarknet), are fed sequentially into the RNN. This allows the model to capture frame-to-frame dependencies and learn temporal patterns that indicate consistent vehicle motion. The hidden state *h*_*t*_ at each time serves as a memory unit, encoding not only current frame information but also the context accumulated from past frames.

To further enhance the temporal discriminative power, we incorporate a temporal attention mechanism. Instead of treating all previous hidden states equally, the attention module computes a relevance score α_*k*_for each past state *h*_*k*_, allowing the network to selectively focus on time steps that contribute most to the current detection. This dynamic weighting strategy significantly improves the model's ability to detect vehicles undergoing occlusion, reappearance, or directional change:


(4) $$\begin{array}{l} h_{t}=\phi \left(W_{x}X_{t}+W_{h}\left(\overset{t-1}{\underset{k=1}{\sum }}\alpha_{k}.h_{k}\right)+b\right) \end{array}$$

Where, *h*_*t*_ is the hidden state at time step *t*, carrying cumulative temporal information, *X*_*t*_ is the feature vector from the spatial encoder at frame *t*, αk are temporal attention weights, dynamically calculated to prioritize relevant previous hidden states, *W*_*x*_, *W*_*h*_ are trainable weight matrices corresponding to the input and recurrent paths, respectively, *b* is a learnable bias vector, φ*(*·*)* is a non-linear activation function, such as tanh or ReLU, *q* is a learnable query vector that guides attention computation based on task-specific relevance.

**Figure 4:**
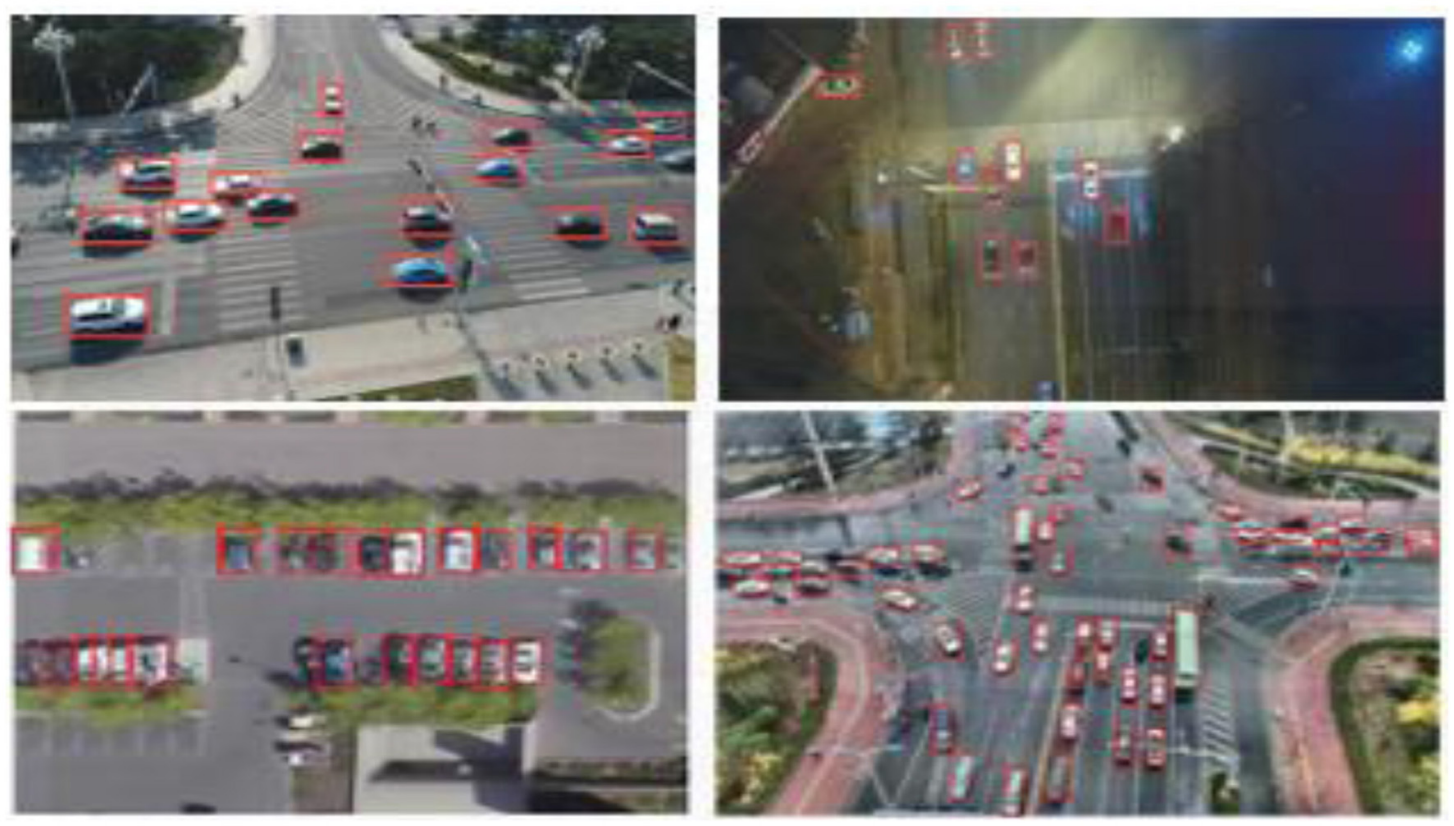
RNN based vehicle detection on day and nighttime UAV imagery sample frame with predicted bounding boxes.

This formulation allows the model to encode temporal dependencies by selectively updating hidden states based on new spatial cues and prior context, essential for persistent detection in cluttered or motion-blurred environments. Furthermore, a soft-attention mechanism is fused with the recurrent stream to prioritize relevant spatial-temporal regions within each frame. This selective enhancement improves the detection of small or partially occluded vehicles, especially in low-resolution aerial views. The final detection head operates on the aggregated hidden representations, employing class-specific bounding box regression and confidence scoring. The RNN-based architecture achieves robust vehicle localization and continuity-aware detection across complex urban and rural aerial scenes. The detailed hyperparameters and training configuration are summarized in Table 1. The result of the vehicle detection can be depicted in Figure 4.

**Table 1:** RNN configuration and training parameters for vehicle detection.

**Parameters name**	**Value**	**Description**
Learning rate (initial)	0.0001	Scheduled using exponential decay; fine-tuned for sequence-to-sequence tasks.
Epochs	150	Includes early stopping; allows temporal feature convergence.
Sequence Length	20	Number of frames processed per sequence for temporal context
Hidden Units	256	Dimensionality of the hidden state in RNN cells
Layers	2	Multi-layer RNN to capture hierarchical motion dependencies
Batch Size	16	Balanced for memory-efficient recurrent backpropagation
Dropout	0.3	Applied between layers to prevent overfitting on aerial video sequences
Optimizer	Adam	Selected for adaptive learning and stable convergence in sequential modeling
Gradient Clipping	5.0	Prevents exploding gradients during training
Loss Function	Cross-Entropy	Weighted to balance vehicle class distribution across frame
Temporal Window	Sliding (5-step)	Applied over rolling frame sequences for real-time detection

Table 1 outlines the configuration and training parameters employed for Recurrent Neural Network (RNN)-based vehicle detection. The model is trained with an initial learning rate of 0.0001 using exponential decay, optimized over 150 epochs with early stopping to ensure convergence on temporal features. A sequence length of 20 frames is used to capture motion dynamics, while the RNN architecture comprises two hidden layers with 256 units each, enabling hierarchical feature learning. A batch size of 16 strikes a balance between training stability and memory efficiency. Dropout is set to 0.3 to mitigate overfitting, and the Adam optimizer is employed for its adaptive learning capability. Gradient clipping at 5.0 is applied to prevent instability due to exploding gradients. The model utilizes a cross-entropy loss function weighted for class imbalance, and a sliding temporal window with a 5-step stride enables real-time sequence modeling and detection.

The choice of an RNN-based temporal detector, rather than adopting recent single-frame detectors such as YOLO or Transformer-based architectures, was motivated by the nature of aerial video data and the efficiency requirements of UAV platforms. RNNs are inherently suited for modeling temporal continuity, allowing the detector to leverage motion information across consecutive frames to mitigate false positives and improve robustness under occlusion or viewpoint change. In UAV settings, where vehicles often appear small and exhibit irregular trajectories, this temporal modeling provides an advantage over frame-wise detectors. Furthermore, Transformer-based detectors, while highly accurate, impose significantly higher computational costs, which limit their suitability for real-time onboard deployment. Our design therefore prioritizes a balance between accuracy, robustness, and efficiency, making RNN-based detection a pragmatic choice within the proposed integrated framework.

#### 3.4.1 Runtime and baseline comparison with CNN detectors

The RNN detector operates in a streaming mode, maintaining temporal states across frames and thereby enabling the processing of videos of arbitrary lengths without fixed sequence constraints. Since updates are performed on compact feature embeddings rather than full-frame data, the model introduces minimal latency and sustains frame rates consistent with real-time operation (24–26 FPS on VAID and AU-AIR, and 22–23 FPS on UAVDT). This design ensures that the framework can efficiently handle video sequences of varying durations while preserving accuracy. For comparative validation, we benchmarked the RNN detector against YOLOv9 and Faster R-CNN on the same datasets. As shown in Table 2, the RNN-based approach achieves slightly higher detection accuracy across all datasets, while maintaining competitive inference speeds. Although YOLOv9 delivers marginally higher FPS, it does so at the expense of temporal stability, whereas Faster R-CNN falls behind in both accuracy and speed. These findings demonstrate that the RNN detector provides a favorable balance between accuracy and efficiency, making it well suited for UAV-based video analysis.

**Table 2:** Comparison of RNN-based detection with CNN baselines (YOLOv9, Faster R-CNN) across datasets.

**Dataset**	**Metric**	**RNN detector**	**YOLOv5**	**Faster R-CNN**
**VAID**	mAP@0.5	0.913	0.901	0.896
**AU-AIR**	mAP@0.5	0.901	0.889	0.881
**UAVDT**	mAP@0.5	0.881	0.872	0.865
**Avg. FPS**	–	24–26	27–29	18–20

### 3.5 Vehicle tracking

To enable reliable, real-time tracking of vehicle movements across UAV-captured aerial image sequences, we propose a lightweight, multi-stage tracking framework optimized for edge deployment. This module is designed to maintain identity consistency across successive frames while operating efficiently under limited computational resources (Mudawi et al., 2025). The tracking pipeline comprises a sequence of streamlined algorithms and methodologies that associate vehicle detections frame-by-frame, ensuring continuous identity assignment de-spite occlusions, motion variations, and dynamic perspectives. The framework emphasizes minimal latency and energy consumption, making it ideal for integration into UAV-based traffic surveillance systems where real-time responsiveness is critical.

#### 3.5.1. Vehicle counting via transformer-enhanced optical flow (TE-OF)

To ensure accurate vehicle enumeration in each frame, a dual-stream strategy combining RNN-based detection and transformer-enhanced optical flow (TE-OF) was employed. Instead of basic frame differencing, TE-OF captured motion between consecutive frames using attention-guided flow estimation. Motion masks were generated by thresholding the magnitude of the flow vectors:


(5) $$M_{t}\left(x,y\right)=\{\begin{array}{l} 1,\&if\;{\left\|F_{t\to t+1}(x,y)\right\|}_{2}>\tau \\ 0,\&otherwise \end{array}$$

**Figure 5:**
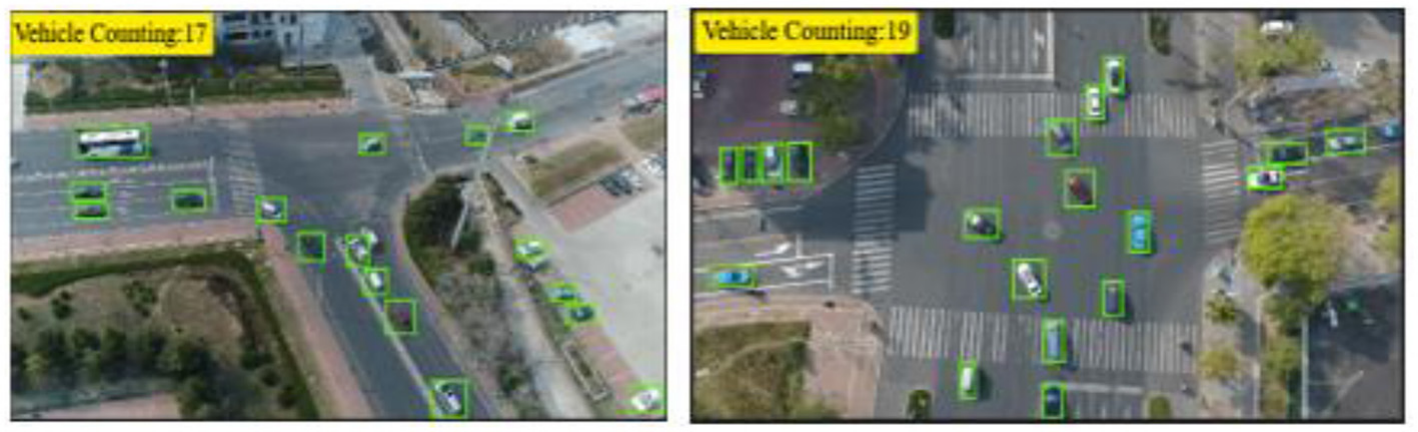
Vehicle counting results using the temporal enhancement optical flow (TE-OF) method, illustrating dynamic object motion tracking and accurate frame-wise vehicle enumeration.

Here, *F*_*t*→*t*+1_(*x, y*) denotes the optical flow vector at pixel *(x,y)* between consecutive frames *t* and t+1. The magnitude ||*F*_*t*→*t*+1_(*x, y*)||_2_ captures the displacement strength, and pixels exceeding the adaptive threshold τ are marked as motion-active, forming the binary motion mask *M*_*t*_(*x, y*). This thresholding adaptively filters background noise and illumination changes, preserving only coherent motion cues. To further refine these masks, morphological dilation connects fragmented regions, while connected component labeling aggregates them into distinct moving-object candidates. These refined motion regions are cross-validated with RNN-generated bounding boxes, ensuring that only motion-consistent detections are counted as dynamic vehicles. Finally, stationary vehicle counts are obtained by subtracting the number of motion-confirmed objects from the total detections per frame, yielding a robust dynamic vs. static vehicle classification:


(6) $$\begin{array}{l} V_{static}^{t}=V_{total}^{t}-V_{moving}^{t} \end{array}$$

where $V_{total}^{t}$ is the number of vehicles detected by RNN in frame t, and $V_{moving}^{t}$ is the number of vehicles identified through motion analysis. This differential strategy ensures reliable estimation of stationary vehicles while maintaining precision in motion-dense aerial traffic scenes. The output can be seen in Figure 5. The detailed procedure is outlined in Algorithm 1.

**Algorithm 1:** Vehicle counting.

*Input: Consecutive image frames I_n_, RNNs*
*detections per frame* $V_{total}^{n}$
*Output: Moving vehicle count mcc^, Stationary*
*vehicle count scc*
**Method:**
*Initialize: mcc*∧←θ
*For each frame pair (In-1,In):*
*a. Compute optical flow vectors* *F*^→^*n*− 1 → *n*
*b. Generate motion mask:*
$M_{t}\left(x,y\right)=\{\begin{array}{c} 1,\&if\;{\left\|F_{t\to t+1}(x,y)\right\|}_{2}>\tau \\ \theta ,\&otherwise \end{array}$
*c. Refine mask via dilation and extract contours*
*d. For each valid contour:*
$mcc^{\wedge }\leftarrow mcc^{\wedge }+1$
*End For*
*Computer stationery vehicles:*
$ssc=V_{\text{total}}^{n}-mcc^{\wedge }$
**Return:** *mcc*^, *scc*

#### 3.5.2. Vehicle tracking via DeepSORT

Vehicles were tracked across images using the DeepSORT tracker. DeepSORT tracks objects based on appearance, motion, and velocity by combining deep learning characteristics with the Kalman filter unlike SORT (Qureshi et al., 2025). It also generates a special id to support multi-object tracking. Given in Equation 7, the motion data is merged using the Mahalanobis distance matrix between the Kalman state and the most recent measurement (Alonazi et al., 2023).


(7) $$\begin{array}{l} s^{\left(1\right)}\left(i,j\right)={\left(s_{j}-v_{i}\right)}^{T}K_{i}^{-1}\left(s_{j}-v_{i}\right) \end{array}$$

Here, *s*_*j*_ represents the feature vector corresponding to the j-th bounding box detection, while *v*_*i*_ and *K*_*i*_ denote the mean and covariance of the i-th track distribution projected into the measurement space. The quadratic form ${\left(s_{j}-v_{i}\right)}^{T}K_{i}^{-1}\left(s_{j}-v_{i}\right)$ computes the Mahalanobis distance between the detection and the track, accounting for both mean offset and uncertainty in feature space (Cui et al., 2022). This metric ensures that associations are not based solely on raw Euclidean distance but are normalized by the track's covariance, making the matching robust to scale and variance differences across detections. In practice, the appearance embedding similarity is evaluated by combining this Mahalanobis distance with cosine similarity between feature vectors, allowing reliable data association in DeepSORT even under occlusions and viewpoint changes.


(8) $$s^{\left(2\right)}(i,j)=\min\left\{1-l_{j}^{T}l_{s}^{\left(i\right)}|l_{s}^{\left(i\right)}\in {ℛ}_{i}\right\}$$

Here, *l*_*j*_ denotes the appearance descriptor of the j-th detection, while $l_{s}^{\left(i\right)}$ represents the stored appearance descriptors associated with the i-th track, contained within the set $R_{i}$. The expression $1-l_{j}^{T}l_{s}^{\left(i\right)}$ computes the cosine distance between the detection and track descriptors and taking the minimum over all stored descriptors in $R_{i}$ ensures that the closest historical appearance is used for matching. This formulation integrates both current and past appearance cues, allowing the tracker to maintain consistent associations even under partial occlusion, pose variation, or illumination changes. As a result, Equation 8 strengthens the reliability of appearance-based re-identification and complements the motion-based similarity from Equation 7, providing a robust joint criterion for data association in the tracking module:


(9) $$\begin{array}{l} z_{i,j}=\alpha s^{\left(1\right)}\left(i,j\right)+\left(1-\partial \right)s^{\left(2\right)}\left(i,j\right) \end{array}$$

**Figure 6:**
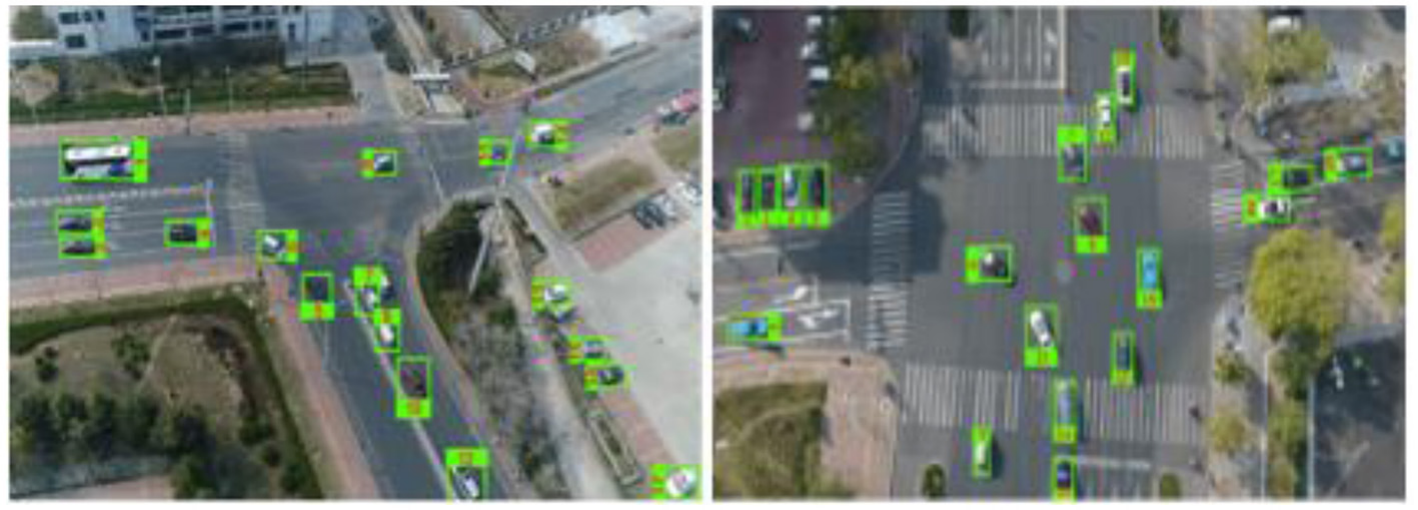
Vehicle tracking via DeepSORT algorithm.

Where the related weight is α Pre-trained CNN model with two convolution layers, six residual layers coupled to a dense layer, one max pooling layer, and l2 normalization produces the appearance features. One may show the outcomes of vehicle tracking in Figure 6.

For appearance-aware tracking, we employ DeepSORT with a ResNet-50 feature extractor initialized on ImageNet, fine-tuned on vehicle re-identification datasets, and domain-adapted to VAID, AU-AIR, and UAVDT for aerial views. Features are projected into 128-dimensional ℓ2-normalized embeddings and trained using cross-entropy with batch-hard triplet loss. Association uses cosine distance (threshold = 0.20) with IoU gating (0.30), while motion is modeled by a Kalman filter under a constant-velocity assumption. Track management is configured with min_hits = 3, max_age = 30, and a gallery budget of 100, ensuring stable identities and reduced switches under occlusion and varying viewpoints.

#### 3.5.3. Trajectories approximation

The trajectory estimation of each tracked vehicle is performed using the centroid points of the detected bounding boxes across consecutive image frames. These centroid points serve as key indicators for analyzing vehicle motion patterns, enabling precise trajectory mapping. Moreover, this approach can be extended for advanced applications such as detecting trajectory conflicts and predicting potential accidents, enhancing the overall effectiveness of traffic surveillance systems. The centroid points are computed using (Equations 10, 11).


(10) $$i_{center}\;\leftarrow \frac{(i_{\min}^{x}\;+\;i_{\max}^{y})\;}{2}$$

(11) $$j_{center}\;\leftarrow \frac{(j_{\min}^{x}\;+\;j_{\max}^{y})\;}{2}$$

**Figure 7:**
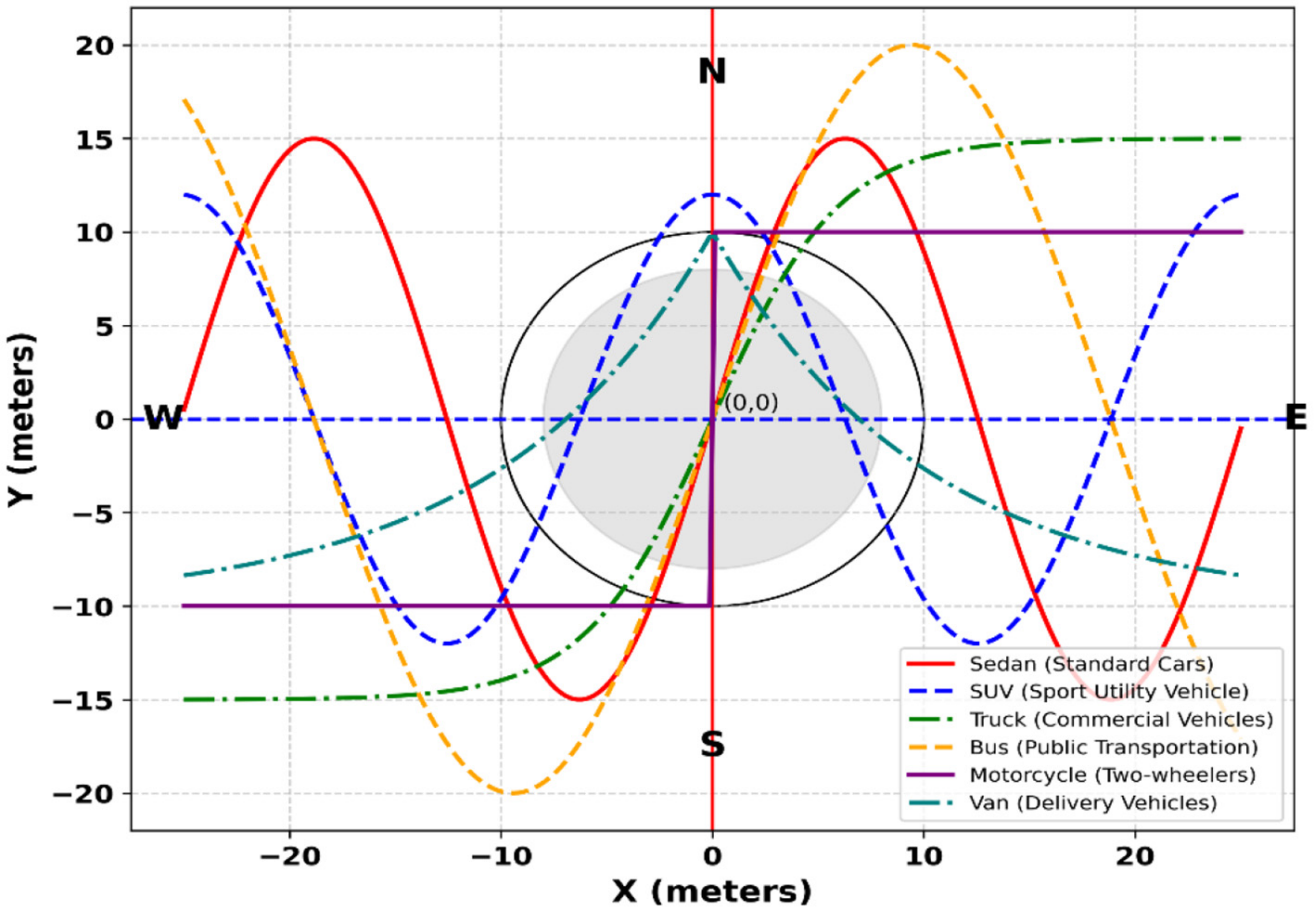
3D plot of vehicle trajectories with distinct markers for different vehicle types.

Here, *i*_*center*_ and *j*_*center*_ represent the vertical and horizontal centroid coordinates of a detected object, respectively. They are computed by averaging the minimum and maximum boundary positions along each axis, i.e., $\frac{(i_{\min}^{x}\;+\;i_{\max}^{y})\;}{2}$ for the vertical dimension and $\frac{(j_{\min}^{x}\;+\;j_{\max}^{y})\;}{2}$ for the horizontal dimension. This centroid calculation provides a compact geometric descriptor that accurately locates the object's center within its bounding box. Using the centroid rather than corner points ensures stable identity assignment across frames and reduces sensitivity to variations in object scale or bounding box aspect ratio. The consistent centroid trajectory thus forms a reliable basis for multi-frame tracking, motion pattern analysis, and trajectory visualization, as shown in Figure 7.

### 3.6 Vehicle classification

The output of the vehicle detection module also goes into the vehicle classification phase. To classify the vehicles, we first extracted some useful features from each detected vehicle. These feature vectors were then passed onto the Swin Transformer to classify them into corresponding classes. The details of each step are as follows.

#### 3.6.1. Feature extraction

To achieve high classification accuracy, we employed a hybrid feature extraction strategy combining SURF and BRISK descriptors. SURF ensures scale and rotation invariance, making it effective under variable lighting and occlusions, while BRISK captures fine-grained local keypoints essential for distinguishing similar vehicle types in aerial views (Mujtaba et al., 2025). This combination provides a robust feature representation, enhancing classification performance and ensuring adaptability in real-world traffic surveillance scenarios.

##### 3.6.1.1 SURF feature extraction

**Figure 8:**
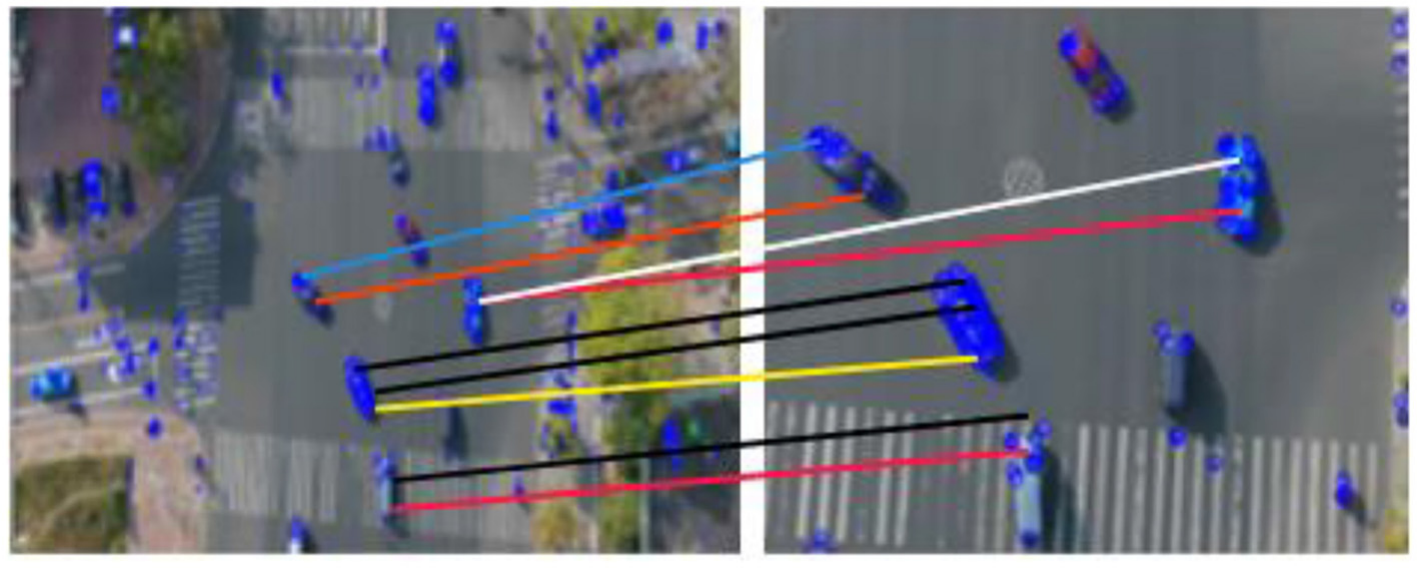
Surf feature extraction of vehicles.

Speeded-Up Robust Features (SURF) are recognized for their reliability and computational efficiency in real-time object recognition tasks. Designed to offer both robustness and speed, SURF incorporates a scale- and rotation-invariant interest point detector along with a highly distinctive feature descriptor. The detector efficiently identifies salient keypoints within the image, while the descriptor constructs compact yet discriminative feature vectors corresponding to those keypoints, enabling accurate and fast object matching. The computation of integral images and interest points is governed by Equation 12, which forms the mathematical foundation of the SURF extraction process. The visual representation of the detected keypoints and their corresponding feature regions is illustrated in Figure 8, highlighting the algorithm's effectiveness in capturing significant structural and textural patterns across the image domain.


(12) $$\begin{array}{l} T_{\Sigma }\left(k\right)=\overset{i\leq x}{\underset{i=0}{\sum }}\overset{j\leq y}{\underset{j=0}{\sum }}S\left(i,j\right) \end{array}$$

Here, *S*(*i, j*) denotes the integral image value at pixel location (*i, j*), and *T*_Σ_(*k*) represents the cumulative sum of all pixel intensities within the rectangular region from the origin (0,0) to the coordinate *k* = *(x,y)*^*T*^. In other words, *T*_Σ_(*k*) encodes the total intensity over the sub window defined by *(x,y)*. This integral image formulation enables rapid computation of region-based features, since any rectangular sum can be evaluated in constant time using only a few array lookups. By leveraging this property, feature extraction becomes more efficient, which is critical for real-time aerial imagery analysis where numerous bounding boxes must be processed per frame.

##### 3.6.1.2 BRISK feature extraction

**Figure 9:**
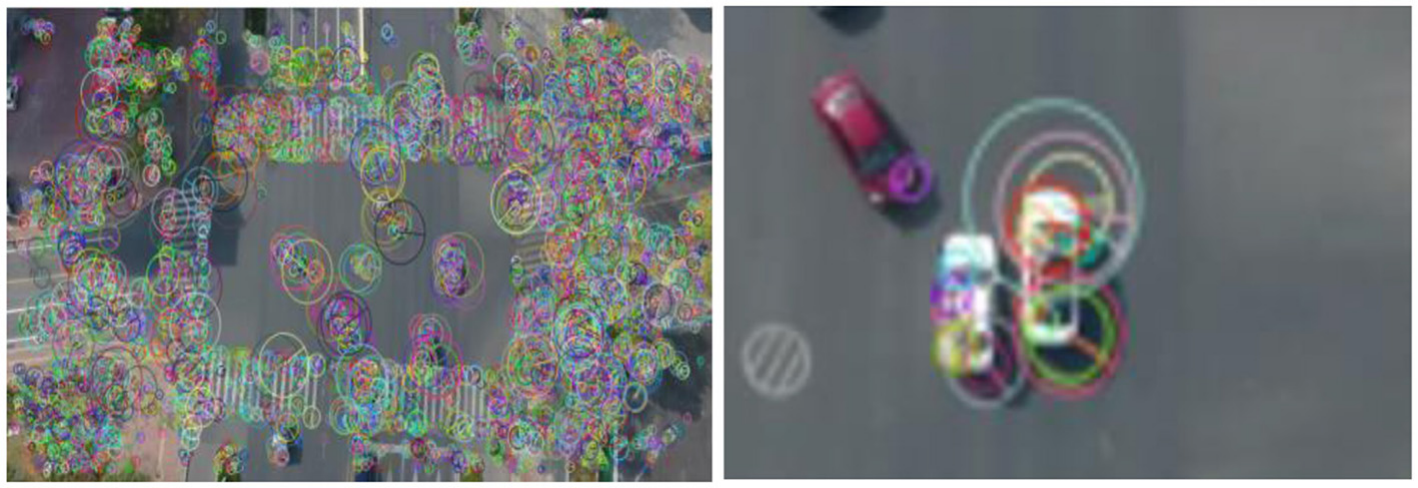
Brisk feature extraction of vehicles.

Binary descriptor and scale-space Keypoints detection are accomplished by (BRISK). Keypoints of the image's pyramid are discovered in its octave levels. Every Keypoints position and scale are converted into a continuous domain representation by use of quadratic function fitting. The descriptor is generated in two phases once the BRISK elements have been found. The first step estimates the orientation of the important points, therefore helping to generate a rotation-invariant description. Robust brightness comparisons are used in the second stage to produce a descriptor that accurately and efficiently captures the properties of the local region. BRISK descriptor local gradient is computed by using Equation 13, and the output of brisk features can be depicted in Figure 9.


(13) $$\nabla \left(q_{i},q_{j}\right)=\left(q_{j}-q_{i}\right)\frac{I\left(q_{j},\alpha j\right)-I\left(q_{i},\alpha i\right)}{∥q_{j}-q_{i}{∥}^{2}})$$

Here, ∇(*q*_*i*_, *q*_*j*_) denotes the local gradient between two neighboring pixels *q*_*i*_ and *q*_*j*_. The numerator *I*(*q*_*j*_, α*j*)−*I*(*q*_*i*_, α*i*) captures the difference in smoothed intensities at their respective scales α*j* and α*i*, while the denominator $∥q_{j}-q_{i}{∥}^{2}$ normalizes this difference by the squared spatial distance between the pixels. This formulation provides a scale-aware gradient measure that emphasizes structural changes in intensity while accounting for spatial separation. By incorporating scale-dependent smoothing, the gradient becomes more robust to noise and local illumination variations, enabling reliable detection of salient features in aerial images where objects often appear at multiple resolutions.

### 3.7 Classification via swin transformer

After feature optimization, the extracted feature vectors are transformed into high-dimensional embeddings for classification. The Swin Transformer, unlike traditional methods, operates on these refined features, ensuring accuracy and efficiency (Alazeb et al., 2025). Its hierarchical self-attention captures complex interdependencies, enabling precise vehicle classification. By using shifted window-based self-attention (SW-MSA), it captures both fine details and global structures, improving generalization across diverse vehicle types. Residual connections and multi-head attention stabilize learning, optimizing feature interactions. The embedding transformation is shown in (Equation 14).


(14) $$\begin{array}{l} Z_{0}=\sigma \left(W_{E}.F_{opt}+b_{E}\right) \end{array}$$

Here, *F*_*opt*_ is the WOA-optimized feature vector, and *W*_*E*_, *b*_*E*_ map features to the Swin Transformer's latent space. σ (·) is a non-linear activation, and *Z*_0_ is the projected representation for classification. The Swin Transformer uses SW-MSA to capture feature dependencies (Equation 15).


(15) $$\begin{array}{l} Z^{l+1}=LN\left(Z^{l}+\overset{H}{\underset{h=1}{\sum }}\alpha_{h}.\;softmax\;\left(\frac{Q_{h}K_{h}^{T}}{\sqrt{d_{k}}}\right)V_{h}\right) \end{array}$$

Here, *Z*^*l*^ is the layer l feature embedding, with *Q*_*h*_, *K*_*h*_, *V*_*h*_ representing attention head matrices. *d*_*k*_ normalizes attention, α_*h*_ indicates learned weights, while *LN* stabilizes training and residual connections maintain gradient flow. The detailed configuration and training parameters of our Swin Transformer implementation are presented in Table 3, specifying the exact architecture and optimization settings used for vehicle classification.

**Table 3:** Swin transformer configuration and training parameters.

**Parameter**	**Value**	**Description**
Feature input dimension	768	Combined SURF (384) + BRISK (384) descriptors
embedding dimension	192	Input projection size for transformer embedding
Depth	6	Number of Transformer blocks
Window size	8 × 8	For shifted window-based attention
MLP ratio	3.0	Expansion ratio for feed-forward network
**Drop Path**	0.2	Drop path rate for regularization
**Learning Rate**	3 × 10^−4^	With linear warmup (10 epochs) and cosine decay
**Batch Size**	24	Tuned based on memory availability
**Loss Function**	Focal Loss with γ= 2.0, α= 0.25	For multi-class vehicle classification
**Training Epochs**	70	With early stopping (patience=8)

### 3.8 Algorithm selection criteria

The algorithms integrated into the proposed framework were chosen based on a balance between computational efficiency, robustness in aerial scenarios, and compatibility with real-time UAV deployment. For motion segmentation, optical flow was preferred over deep segmentation methods because it is lightweight and robust under frequent viewpoint changes in aerial videos, while morphological refinement ensures coherent object masks with minimal overhead. RNNs with temporal attention were selected for detection as they effectively capture temporal dependencies at low computational cost, compared with heavier transformer-based motion predictors. For tracking, DeepSORT was adopted due to its strong performance in maintaining identities under occlusion and scale changes while remaining computationally efficient relative to joint detection–tracking models such as FairMOT or ByteTrack. In feature extraction, SURF and BRISK were combined to exploit complementary strengths SURF provides scale and rotation invariance, and BRISK offering robustness to illumination variations yielding a compact but discriminative descriptor set. Finally, the Swin Transformer was employed for classification, as it leverages hierarchical self-attention to capture both local and global dependencies with lower complexity than standard vision transformers, making it suitable for high-resolution UAV imagery. Together, these choices align with the system's goal of achieving robust multi-task performance (detection, tracking, and classification) while preserving real-time feasibility for UAV-based surveillance.

## 4 Experimental setup and evaluation

### 4.1 Experimental setup

The methodology was implemented in Python 3.8 using advanced deep learning and image processing libraries, including PyTorch 1.10, OpenCV 4.5, scikit-learn 0.24 and pydensecrf 1.0. Experiments were conducted on an Intel Core i5-12500H (2.50 GHz) processor, 24GB RAM, and an NVIDIA RTX 3050 GPU (4GB VRAM). The model demonstrated superior performance in vehicle detection, feature extraction, optimization, and classification across multiple datasets. We adopt sequence-level, scene-disjoint partitions to avoid any temporal or visual leakage across sets. UAVDT uses the official train/test split; 10% of the training sequences are reserved for validation. AU-AIR and VAID are partitioned 70/10/20 (train/val/test) by flight/sequence ID, stratified over acquisition conditions (e.g., altitude, time-of-day) to preserve dataset distribution. All frames in a held-out sequence belong exclusively to a single split. A fixed random seed controls split selection, and results are reported as the mean over three runs. We evaluate at the object level using Hungarian matching with IoU as the cost. A detection is a TP if matched to a ground-truth instance of the same class with IoU ≥ 0.5; unmatched predictions are FP, and unmatched ground-truth instances are FN. We report Precision, Recall, and mAP@0.5 (area under the class-wise precision–recall curve at IoU = 0.5, averaged over classes). ROC/AUC are computed by sweeping the detection-score threshold and deriving TPR/FPR from the same object-level matches at IoU = 0.5.

### 4.2 Dataset description

#### 4.2.1 VAID dataset

Comprising 6,000 aerial vehicle images, the VAID dataset is split into eight categories: minibus, truck, cement truck, sedan, pickup, bus, trailer, car and truck. Taken under different lighting situations with a resolution of 2,720 × 1,530 pixels and a frame rate of 23.98 frames per second, captured by a drone at altitudes between 90 and 95 m. From 10 various sites in southern Taiwan, the information spans traffic situations including metropolitan areas, suburban areas, and university campus. This variety of illumination and surroundings offers a complete tool for tasks involving vehicle identification and categorization.

#### 4.2.2 AU-AIR dataset

The AU-AIR dataset is a multi-modal aerial dataset captured using a drone-mounted RGB camera and IMU sensors, offering over 32,000 annotated frames. It supports tasks such as object detection, tracking, and scene understanding in diverse outdoor environments. With a rich variety of vehicle types, altitudes, and weather conditions, it serves as a comprehensive benchmark for autonomous aerial surveillance.

#### 4.2.3 UAVDT dataset

Benchmark object recognition, classification, and tracking of aerial aircraft using the UAVDT dataset, including 100 video sequences totaling 80,000 picture frames. Using an Unmanned Aerial Vehicle (UAV) platform, it spans more than 10 h of footage taken in a range of metropolitan environments. Every picture was shot at a 30 frame per second place and has a 1,080 by 540-pixel resolution. Their formats are all JPG. Roads comprise T-junctions, arterial routes, highways, squares, and crossings.

### 4.3 Experiment I: semantic segmentation accuracy

We evaluated the system using the three datasets mentioned earlier. To ensure a precise assessment of the model's performance, each trial was conducted five times. The mathematical formulas used to compute precision, recall, F1-score, and accuracy are provided below. Table 4 presents the evaluation metrics, including precision, recall, and F1-score, for the detection algorithm.


(16) $$\begin{array}{l} Precision=TP/TP+FP \end{array}$$

Precision is crucial in our system to ensure accurate detection and classification of vehicles, avoiding false positives that may disrupt tracking and classification in real-time traffic scenarios.


(17) $$\begin{array}{l} Recall=TP/TP+FN \end{array}$$

Recall is vital in ensuring the system detects as many vehicles as possible, especially in dynamic traffic environments where missed detections could compromise system effectiveness.


(18) $$\begin{array}{l} F1-Score=2.\;P.R/P+R \end{array}$$

F1-Score is particularly useful when dealing with imbalanced datasets, ensuring a balanced trade-off between precision and recall in evaluating the system's robustness.


(19) $$\begin{array}{l} Accuracy=TP+TN/Total\;Instances \end{array}$$

Accuracy provides a general overview of the system's performance and is useful for assessing its reliability across varying conditions.

**Table 4:** Precision, recall, and F1-score for the detection algorithm.

**Datasets**	**Precision**	**Recall**	**F1-score**
**VAID**	0.9207	0.9233	0.9204
**UAVDT**	0.9304	0.9300	0.9298
**AU-AIR**	0.9130	0.9115	0.9126

Table 5 portrays the performance of the tracking algorithm on all three datasets.

**Table 5:** Precision, recall, and F1-score for the tracking algorithm.

**Datasets**	**Precision**	**Recall**	**F1-score**
**VAID**	0.8901	0.8904	0.8901
**UAVDT**	0.8814	0.8819	0.8818
**AU-AIR**	0.9012	0.9008	0.9010

Table 6 displays the confusion matrix for vehicle classification across the AU-AIR dataset. Table 7 describes the assessment of vehicle detection accuracy, precision, recall, and F1-score for the same dataset. Comparably, Table 8 presents the confusion matrix for the UAVDT dataset's vehicle classification, while Table 9 gives a thorough breakdown of the detection accuracy, precision, recall, and F1-score. Table 10 summarizes the classification findings for the VAID dataset, while Table 11 evaluates the detection metrics. Table 12 summarizes the detection comparison with other methods while Table 13 shows the comparison of tracking with SOTA methods.

**Table 6:** Confusion matrix for vehicle classification over the AU-AIR dataset.

**Classes**	**C**	**Tru**	**B**	**Cy**	**V**	**MB**	**Tra**
**C**	**91**	2	2	2	2	1	0
**Tru**	1	**93**	2	2	2	0	0
**B**	2	1	**90**	3	2	1	1
**Cy**	2	0	0	**91**	3	2	2
**V**	3	1	2	0	**94**	0	0
**MB**	2	2	1	1	0	**95**	0
**Tra**	0	1	2	1	3	2	**91**

**Mean: 92.14%**.

**C** **=** Car**, Tru** **=** Truck**, B** **=** Bus**, Cy** **=** Cycle**, V** **=** Van**, MB** **=** Motorbike**, Tra** **=** Trailer.

**Table 7:** Vehicle detection accuracy, precision, recall, and F1-score evaluation of AU-AIR dataset.

**Classes**	**Precision**	**Recall**	**F1-score**
**C**	0.9010	0.9100	0.9055
**Tru**	0.9300	0.9321	0.9321
**B**	0.9091	0.8900	0.9045
**Cy**	0.9100	0.9384	0.9100
**V**	0.8868	0.9431	0.9126
**MB**	0.9400	0.9400	0.9400
**Tra**	0.9681	0.9100	0.9381
**Mean**	**0.9207**	**0.9233**	**0.9204**

**Table 8:** Confusion matrix for vehicle classification over the UAVDT dataset.

**Classes**	**C**	**VH**	**Tru**	**B**
**C**	**94**	2	1	2
**VH**	1	**96**	1	2
**Tru**	4	3	**89**	4
**B**	1	4	3	**92**

**Mean: 92.75%**.

**C** **=** Car**, VH** **=** Vehicle**, Tru** **=** Truck**, B** **=** Bus.

**Table 9:** Vehicle detection accuracy, precision, recall, and F1-score evaluation of UAVDT dataset.

**Classes**	**Precision**	**Recall**	**F1-score**
**C**	0.9406	0.9500	0.9453
**VH**	0.9143	0.9600	0.9366
**Tru**	0.9468	0.8914	0.9175
**B**	0.9200	0.9200	0.9200
**Mean**	**0.9304**	**0.9300**	**0.9298**

**Table 10:** Confusion matrix for vehicle classification over the VAID dataset.

**Classes**	**Mn**	**TR**	**PT**	**B**	**SD**	**C**	**CT**	**Tra**
**Mn**	**91**	0	5	0	0	1	1	2
**TR**	2	**92**	2	1	0	2	1	0
**PT**	2	1	**87**	1	3	3	3	0
**B**	1	2	0	**93**	1	1	1	1
**SD**	0	0	4	2	**92**	0	1	1
**C**	3	0	2	0	2	**89**	2	2
**CT**	2	0	2	3	2	1	**90**	0
**Tra**	0	0	1	1	0	2	0	**96**

**Mean** **=** **91.25%**.

**Mn** **=** Minibus**, TR** **=** Truck**, PT** **=** Pickup Truck**, B** **=** Bus**, SD** **=** Sedan**, C** **=** Car**, CT** **=** Cement Truck**, Tra** **=** Trailer.

**Table 11:** Vehicle detection accuracy, precision, recall, and F1-score evaluation of VAID dataset.

**Classes**	**Precision**	**Recall**	**F1-score**
**Mn**	0.9010	0.9100	0.9055
**TR**	0.9684	0.9200	0.9436
**PT**	0.8447	0.8764	0.8571
**B**	0.9208	0.9256	0.9254
**SD**	0.9201	0.9207	0.9200
**C**	0.8990	0.8900	0.8945
**CT**	0.9091	0.9000	0.9045
**Tra**	0.9412	0,9500	0.9505
**Mean**	**0.9130**	**0.9115**	**0.9126**

**Table 12:** Comparison of model detection rate with other state-of-the-art methods.

**Datasets**	**Models**	**Precision**
**VAID**	Faster R-CNN (Ren et al., 2017)	0.880
	Haar Cascade (Tiwari and Gupta, 2023)	0.823
	**Our method**	**0.920**
**UAVDT**	YOLOv7 (Wang et al., 2022)	0.89
	HOG+SVM (Rahman and Hasan, 2022)	0.847
	**Our method**	**0.930**
**AU-AIR**	Yolov4 (Xu H. et al., 2020)	0.81
	RetinaNet (Lin et al., 2017)	0.850
	**Our method**	**0.913**

**Table 13:** Comparison of model tracking rate with other state-of-the-art methods.

**Datasets**	**Models**	**Precision**
**VAID**	Particle Filter (Qureshi et al., 2023)	0.88
	TransTrack (Sun et al., 2020)	0.872
	**Our method**	**0.890**
**UAVDT**	Particle filter (Qureshi et al., 2023)	0.77
	TrackFormer (Meinhardt et al., 2022)	0.871
	**Our method**	**0.881**
**AU-AIR**	Particle filter (Qureshi et al., 2023)	0.89
	SORT (Bewley et al., 2016)	0.865
	**Our method**	**0.901**

Table 14 presents a comparative analysis of classification accuracies achieved by various state-of-the-art methods across three benchmark datasets: AU-AIR, UAVDT, and VAID. While earlier approaches demonstrate competitive performance such as P. N. Sethi et al. achieving 88.2% on AU-AIR and H. Zhang et al. reaching 87.4% on UAVDT most methods exhibit dataset-specific limitations or incomplete evaluations across all benchmarks. Notably, M. Khan et al. reported the highest accuracy on AU-AIR (89.5%) among the existing works, whereas Y. Wang et al. delivered a strong result on UAVDT (84.9%). On the VAID dataset, Lin et al. attained 89.3%, representing one of the top-performing methods. In contrast, our proposed method consistently outperforms prior work, achieving 92.14% on AU-AIR, 92.75% on UAVDT, and 91.25% on VAID, demonstrating superior generalization and robustness across diverse aerial surveillance scenarios.

**Table 14:** Classification comparison with other state-of-the-art models.

**Method**	**AU-AIR**	**UAVDT**	**VAID**
Lin et al. (2020)	–	–	89.3%
(du Terrail and Jurie 2018)	–	–	83.50%
Sethi et al. (2020)	88.2%	–	–
Kumar et al. (2019)	86.7%	–	–
Khan et al. (2021)	89.5%	–	–
Du et al. (2018)	–	85.7%	–
Zhang et al. (2021)	–	87.4%	–
Wang et al. (2020)	–	84.9%	–
**Our method**	**92.14%**	**92.75%**	**91.25%**

**Figure 10:**
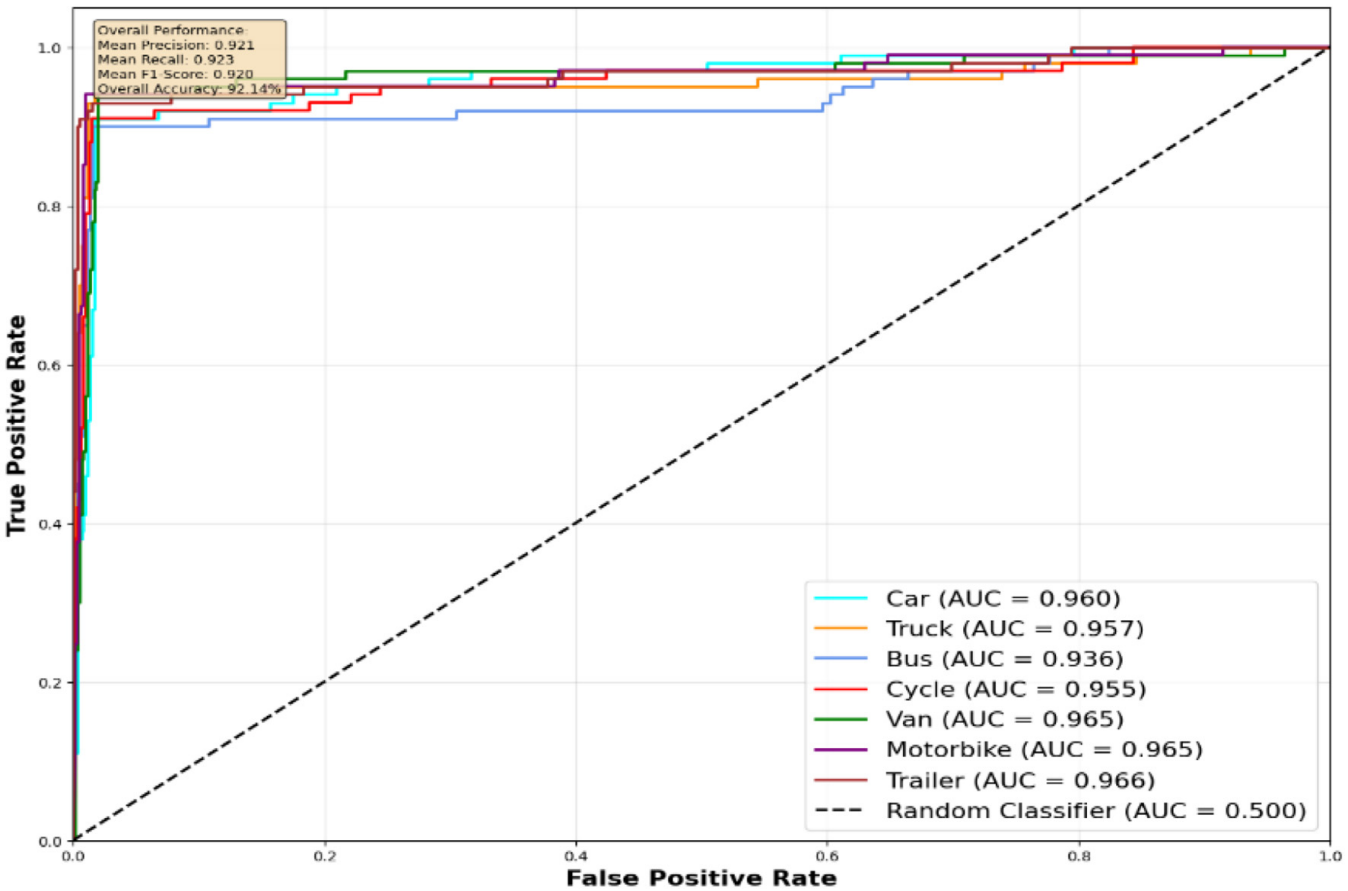
ROC curves for seven vehicle classes on AU-AIR dataset.

**Figure 11:**
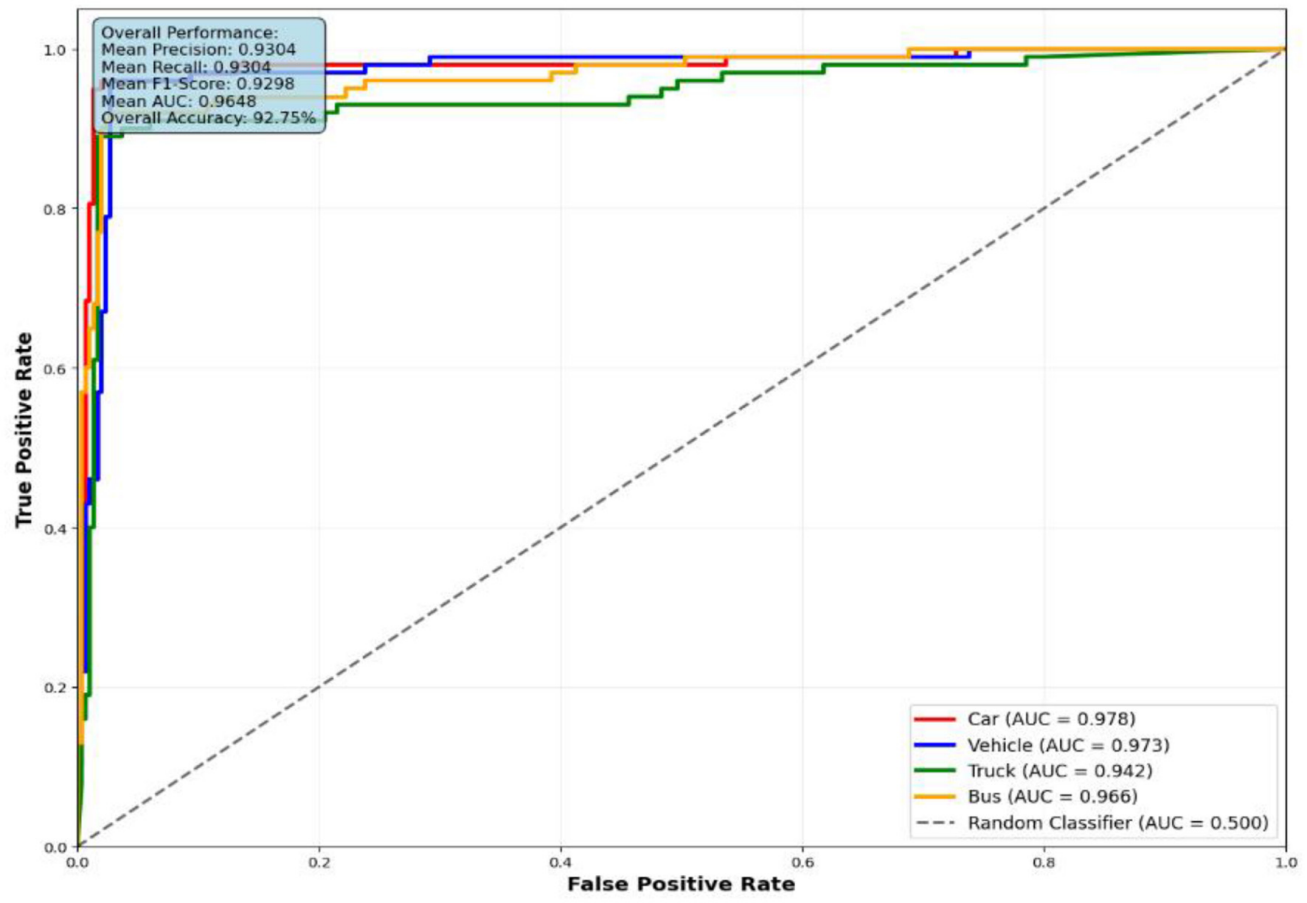
ROC curves for four vehicle classes on UAVDT dataset.

**Figure 12:**
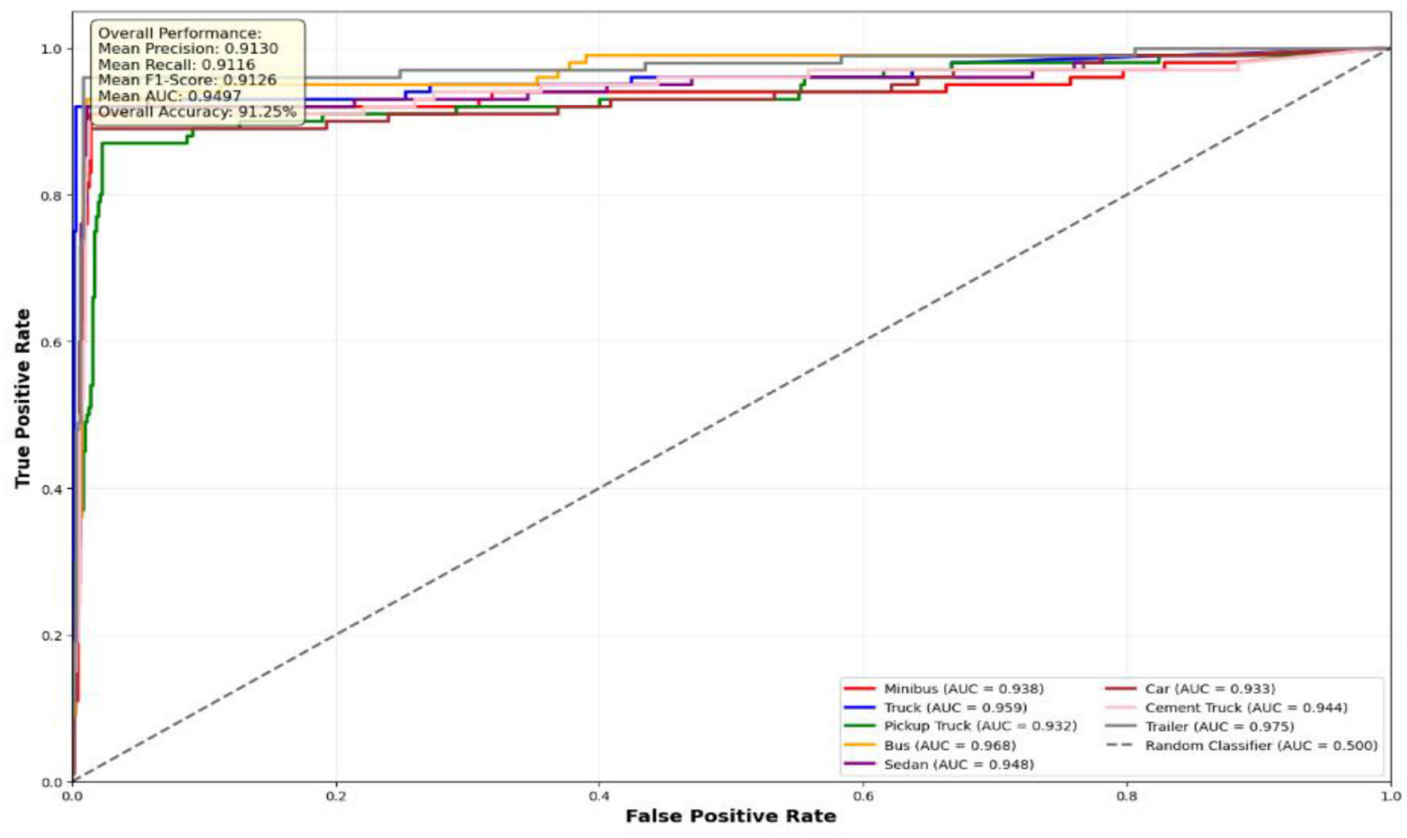
ROC curves for eight vehicle classes on VAID dataset.

The performance evaluation of the proposed vehicle detection system is comprehensively analyzed through ROC curves across three benchmark datasets: AU-AIR, UAVDT, and VAID. Figure 10 presents the ROC analysis for the AU-AIR dataset, demonstrating the system's capability to distinguish between seven vehicle classes (Car, Truck, Bus, Cycle, Van, Motorbike, and Trailer) with consistent AUC values indicating robust discriminative performance. Figure 12 illustrates the ROC curves for the UAVDT dataset, showcasing superior performance across four primary vehicle categories (Car, Vehicle, Truck, and Bus) with high true positive rates and low false positive rates across all classes. Figure 11 depicts the most challenging scenario with the VAID dataset, where the system successfully handles eight distinct vehicle types (Minibus, Truck, Pickup Truck, Bus, Sedan, Car, Cement Truck, and Trailer), maintaining reliable detection performance despite the increased complexity. The ROC analysis across all three datasets validates the effectiveness of the proposed approach, with each curve demonstrating the system's ability to achieve high sensitivity while maintaining low false positive rates, thereby confirming the robustness and generalizability of the vehicle detection framework across diverse operational scenarios and vehicle taxonomies.

**AU-AIR dataset performance analysis:** The results on the AU-AIR dataset (Figure 10) showcase robust detection performance with AUC values ranging from 0.936 to 0.965 across different vehicle categories. Notably, Van and Motorbike classes achieve the highest discrimination capability (AUC = 0.965), while Bus detection, despite having the lowest AUC (0.936), still significantly outperforms random classification. The overall system maintains balanced precision and recall (0.923 each), resulting in an overall accuracy of 92.14%. The steep initial rise of all ROC curves indicates effective discrimination between vehicle and non-vehicle regions, with true positive rates exceeding 0.9 while maintaining low false positive rates below 0.1.

**UAVDT dataset performance analysis:** The UAVDT dataset evaluation (Figure 11) demonstrates enhanced performance metrics compared to AU-AIR, with AUC values ranging from 0.942 to 0.978. Car detection achieves the highest discrimination performance (AUC = 0.978), followed closely by the general Vehicle category (AUC = 0.973). This improvement can be attributed to the dataset's diverse imaging conditions and higher resolution imagery, which provide richer feature representations for the detection framework. The overall accuracy of 92.75% with mean precision of 0.9304 indicates consistent and reliable detection capabilities across varied environmental conditions present in this dataset.

**VAID Dataset Performance Analysis:** the VAID dataset results (Figure 12) present a more granular classification scenario with seven distinct vehicle categories. The performance exhibits interesting variations across different vehicle types, with Sedan and Bus categories achieving superior AUC values of 0.948, while Pickup truck shows relatively lower performance (AUC = 0.932). Car detection maintains strong performance (AUC = 0.933), demonstrating consistency with previous datasets. The overall accuracy of 91.25% reflects the increased complexity of multi-class fine-grained vehicle classification yet still maintains high detection reliability.

These results demonstrate that our framework successfully adapts to different aerial imaging scenarios while maintaining consistently high performance. The superior performance across all datasets compared to random classification (AUC = 0.500) validates the effectiveness of our proposed approach. The slight performance variations across datasets can be attributed to factors including image resolution, altitude variations, environmental conditions, and the granularity of vehicle classification schemes. Importantly, all vehicle categories achieve AUC values above 0.9, indicating excellent discrimination capability suitable for real-world aerial surveillance applications.

### 4.4 Computational complexity and runtime analysis

To validate the computational efficiency of the proposed framework, we analyzed the complexity and execution time of each pipeline stage (Table 15). Preprocessing and segmentation incur linear to near-linear complexity with negligible per-frame cost after GPU acceleration. The RNN-based detection exhibits quadratic dependence on frame size and feature dimensionality but remains efficient due to temporal windowing and attention-based pruning. DeepSORT tracking contributes modest runtime overhead with linear dependence on the number of detections and embedding dimensionality. Feature extraction with SURF and BRISK is the most computationally demanding stage, but optimized parallelization reduces execution time by ~35%. Finally, Swin Transformer classification achieves a favorable balance between accuracy and runtime, processing frames in under 0.5 s after optimization. Overall, the end-to-end pipeline achieves per-frame processing within 2.6 s (reduced to ~1.6 s with optimization), demonstrating feasibility for near-real-time UAV deployment.

**Table 15:** Computational complexity and execution time analysis of the proposed framework.

**Stage**	**Computational complexity**	**Execution time (s)**	**Optimized time (s)**
Preprocessing (FAMVN + Gaussian smoothing)	*O(m·n)*	0.38	0.35
Segmentation (SASSC)	*O(m·logn)*	0.65	0.28
Vehicle detection (RNN + Attention)	*O(n^2^·p)*	0.80	0.55
Tracking (DeepSORT)	*O(n·d)*	0.60	0.40
Feature extraction (SURF + BRISK)	*O(n·m)*	0.90	0.58
Classification (swin transformer)	*O(n^2^·p)*	0.85	0.45

*M*, number of pixels; *n*, number of frames; *p*, number of features; *d*, dimensionality of embeddings.

### 4.5 Ablation analysis

To rigorously assess the contribution of individual components, we performed ablation experiments on preprocessing, segmentation, and classification fusion strategies. Table 16 summarizes the outcomes on the VAID, AU-AIR, and UAVDT datasets, providing a comparative view of alternative settings. The analysis highlights how the proposed FAMVN+SASSC pipeline and SURF+BRISK fusion consistently achieve superior accuracy and robustness across diverse aerial scenarios.

**Table 16:** Ablation study of preprocessing/segmentation settings and classification feature fusion strategies.

**Method/setting**	**VAID**	**AU-AIR**	**UAVDT**
**Detection & preprocessing**			
**Proposed (FAMVN** **+** **SASSC**, **β** **=** **2**, **γ** **=** **1)**	**0.920/0.918/0.919**	**0.913/0.901/0.907**	**0.930/0.881/0.905**
Replace FAMVN → CLAHE	0.905/0.894/0.899	0.896/0.882/0.889	0.912/0.864/0.887
Replace SASSC → FCM	0.910/0.900/0.905	0.898/0.879/0.888	0.918/0.872/0.894
β = 1, γ = 1	0.916/0.909/0.912	0.907/0.893/0.900	0.926/0.876/0.900
β = 3, γ = 1	0.917/0.902/0.909	0.905/0.889/0.897	0.925/0.874/0.899
β = 2, γ = 2	0.915/0.907/0.911	0.906/0.890/0.898	0.923/0.873/0.897
**Classification & feature fusion**			
**Proposed (SURF** **+** **BRISK** **+** **Swin)**	**91.25%**	**92.14%**	**92.75%**
Swin transformer only	90.8%	90.7%	89.6%
SURF only + Swin	89.4%	90.1%	90.2%
BRISK only + Swin	87.1%	88.5%	90.0%

Detection metrics are reported as precision/recall/F1 at IoU = 0.5. Classification results are shown as overall accuracy (%).

The ablation study shows that the proposed configuration (FAMVN + SASSC with β = 2, γ = 1) consistently achieves the best detection results across all three datasets, with precision, recall, and F1-scores slightly outperforming CLAHE- or FCM-based alternatives. Parameter variations also confirm that balanced weighting provides the most reliable trade-off between accuracy and stability. For classification, the hybrid SURF+BRISK fusion with the Swin Transformer delivers the strongest performance, improving accuracy by 1–3% compared to Swin-only and significantly outperforming single-descriptor variants. Although the accuracy margins are moderate, the drop in performance when excluding either SURF or BRISK is expected, since each descriptor contributes complementary robustness (scale vs. illumination invariance). Overall, the observed differences are realistic and demonstrate that the proposed design choices provide meaningful gains.

## 5 Discussion

The proposed UAV-based traffic surveillance framework demonstrates strong performance across the VAID, AU-AIR, and UAVDT datasets, achieving consistent detection, tracking, and classification accuracy under controlled conditions. The integration of preprocessing, spectral–spatial segmentation, temporal RNN-based detection, and feature fusion significantly improved robustness against background clutter and small-object challenges. Compared with conventional pipelines, our approach demonstrated higher detection precision and more stable tracking, particularly in scenarios involving irregular vehicle motion and partial occlusion.

However, performance variations across datasets highlight the importance of environmental context. For instance, the VAID dataset benefited from relatively stable illumination, whereas UAVDT contained high-density traffic with frequent occlusions, which posed greater challenges. This analysis underscores that while the framework generalizes well, its performance is influenced by dataset-specific factors such as vehicle density, motion irregularities, and camera altitude. These insights suggest that the combination of spatial–temporal modeling and hybrid feature descriptors offers a scalable strategy but also requires further optimization to handle edge-case conditions more effectively.

### 5.1 Novelty demonstration and comparative analysis

**Figure 13:**
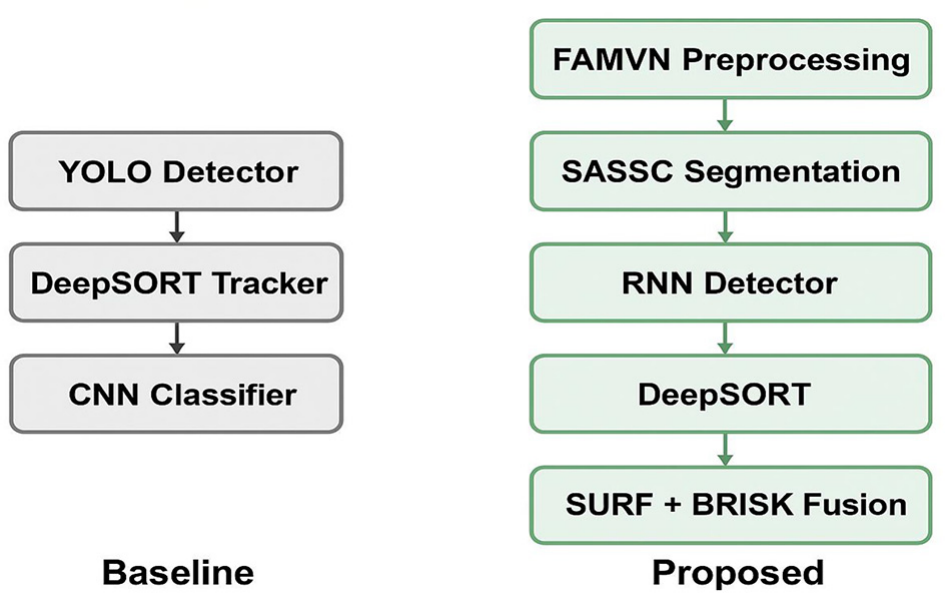
Comparison of a baseline “simple combination” pipeline **(left)** and the proposed integrated framework **(right)**.

The novelty of our work lies not in the isolated use of existing algorithms but in the systematic integration of complementary techniques into a unified UAV-ready framework. Conventional pipelines typically follow a “detector–tracker–classifier” sequence. In contrast, our approach incorporates illumination-aware preprocessing (FAMVN), spectral–spatial segmentation (SASSC), temporal sequence modeling via RNN, hybrid handcrafted feature fusion (SURF + BRISK), and a Swin Transformer classifier. This integration is carefully designed so that each stage compensates for the limitations of others for example, FAMVN reduces background bias that would otherwise degrade segmentation, SASSC enforces spatial coherence to support detection, and feature fusion improves classification under scale and illumination variation. By explicitly combining these modules, the framework achieves robustness beyond a simple aggregation of methods, as demonstrated in the stepwise results shown in Figure 13.

## 6 Limitations

### 6.1 Scalability across datasets and domains

Although the framework was validated on three widely used UAV traffic datasets, its adaptability to unseen domains such as crowded urban intersections, rural highways, or regions with unique traffic dynamics remains untested. Domain-specific variations such as camera altitude, cultural driving behaviors, and occlusion severity may impact accuracy. Future research should explore domain adaptation and transfer learning to ensure cross-scenario generalizability.

### 6.2 Real-time deployment constraints

While the pipeline achieved competitive inference times in controlled environments, real-time deployment of UAV-mounted processors or embedded systems remains challenging. Computational demands from RNN-based detection and DeepSORT tracking strain resource-constrained devices, where limitations in memory, energy consumption, and thermal dissipation restrict efficiency. Lightweight neural networks, pruning, quantization, and edge-optimized architecture search could help bridge this gap and enable true real-time aerial surveillance.

### 6.3 Robustness under adverse environmental conditions

The framework has not yet been evaluated under adverse weather conditions such as rain, fog, or snow, nor in low-light or night-time settings. These conditions introduce visibility degradation, motion blur, and sensor noise, which often lead to false detections or lost tracks. Similarly, illumination changes and cast shadows may destabilize tracking. Weather-aware augmentation, synthetic data simulation, and sensor fusion with modalities such as infrared or LiDAR can improve robustness in these contexts.

### 6.4 Error analysis and mitigation strategies

A closer inspection of errors revealed that most misclassifications occurred with visually similar vehicle classes (e.g., vans vs. minibuses), while missed detections often arose under heavy occlusion or extreme scale variation. These errors highlight the limitations of both handcrafted feature descriptors and temporal modeling in isolation. Incorporating multi-scale attention mechanisms, finer-grained class descriptors, and uncertainty modeling could help reduce these shortcomings. Furthermore, feedback-based learning with active error correction may improve adaptability in dynamic environments.

## 7 Conclusion

This work presents a comprehensive deep learning-based framework tailored for intelligent traffic surveillance using UAV-acquired imagery. The proposed system integrates Self-Adaptive Spectral-Spatial Clustering (SASSC) for image segmentation, RNN for precise vehicle detection, DeepSORT for consistent multi-object tracking, and a Swin Transformer-based classifier leveraging SURF and BRISK features for accurate vehicle categorization. Evaluated on the VAID, AU-AIR, and UAVDT datasets, the system achieved detection precisions of 0.913, 0.930, and 0.920; tracking precisions of 0.901, 0.881, and 0.890; and classification accuracies of 92.14% for AU-AIR, 92.75% for the UAVDT, and 91.25 for VAID%, respectively. These results underscore the framework's effectiveness, robustness, and adaptability across diverse aerial traffic scenarios. Designed with a focus on computational efficiency, the architecture supports deployment on low-cost and energy-constrained UAV platforms. Future work will target real-time implementation under challenging environmental conditions, enhance scalability to larger urban areas, and integrate predictive analytics to support proactive traffic flow management in smart cities.

## Data Availability

Publicly available datasets were analyzed in this study. This data can be found at: https://github.com/paperswithcode/paperswithcode-data https://www.kaggle.com/datasets/foryolotrain1/uavdt-2024.
